# Deciphering the Transcriptional-Regulatory Network of Flocculation in *Schizosaccharomyces pombe*

**DOI:** 10.1371/journal.pgen.1003104

**Published:** 2012-12-06

**Authors:** Eun-Joo Gina Kwon, Amy Laderoute, Kate Chatfield-Reed, Lianne Vachon, Jim Karagiannis, Gordon Chua

**Affiliations:** 1 Institute for Biocomplexity and Informatics, University of Calgary, Calgary, Alberta, Canada; 2 Department of Biological Sciences, University of Calgary, Calgary, Alberta, Canada; 3 Department of Biology, University of Western Ontario, London, Ontario, Canada; University of Michigan, United States of America

## Abstract

In the fission yeast *Schizosaccharomyces pombe*, the transcriptional-regulatory network that governs flocculation remains poorly understood. Here, we systematically screened an array of transcription factor deletion and overexpression strains for flocculation and performed microarray expression profiling and ChIP–chip analysis to identify the flocculin target genes. We identified five transcription factors that displayed novel roles in the activation or inhibition of flocculation (Rfl1, Adn2, Adn3, Sre2, and Yox1), in addition to the previously-known Mbx2, Cbf11, and Cbf12 regulators. Overexpression of *mbx2^+^* and deletion of *rfl1^+^* resulted in strong flocculation and transcriptional upregulation of *gsf2* ^+^/*pfl1^+^* and several other putative flocculin genes (*pfl2^+^–pfl9^+^*). Overexpression of the *pfl^+^* genes singly was sufficient to trigger flocculation, and enhanced flocculation was observed in several combinations of double *pfl^+^* overexpression. Among the *pfl1^+^* genes, only loss of *gsf2^+^* abrogated the flocculent phenotype of all the transcription factor mutants and prevented flocculation when cells were grown in inducing medium containing glycerol and ethanol as the carbon source, thereby indicating that Gsf2 is the dominant flocculin. In contrast, the mild flocculation of *adn2^+^* or *adn3^+^* overexpression was likely mediated by the transcriptional activation of cell wall–remodeling genes including *gas2^+^*, *psu1^+^*, and SPAC4H3.03c. We also discovered that Mbx2 and Cbf12 displayed transcriptional autoregulation, and Rfl1 repressed *gsf2^+^* expression in an inhibitory feed-forward loop involving *mbx2^+^*. These results reveal that flocculation in *S. pombe* is regulated by a complex network of multiple transcription factors and target genes encoding flocculins and cell wall–remodeling enzymes. Moreover, comparisons between the flocculation transcriptional-regulatory networks of *Saccharomyces cerevisiae* and *S. pombe* indicate substantial rewiring of transcription factors and cis-regulatory sequences.

## Author Summary

Flocculation is a process that involves yeast cells adhering to one another to form clumps called flocs. This trait is important for industrial yeast applications as it provides a cost-effective and efficient method to remove yeast cells. The adherence between cells occurs by the binding of glycoproteins known as flocculins and carbohydrate molecules located on the cell surface. To better understand how flocculation works, the genes that encode for flocculins and the transcription factors that regulate their expression need to be identified. In the fission yeast *S. pombe*, many of the flocculins and transcription factors that function in flocculation are not known. To address this gap in knowledge, we have employed molecular genetics and functional genomic approaches to uncover transcription factors and their target genes that play a role in flocculation. We discover that flocculation in *S. pombe* is regulated by a complex network of transcription factors that activate and repress themselves, as well as multiple target genes that encode for flocculins and cell wall–remodeling enzymes. The comparison of the flocculation regulatory networks between fission and budding yeasts indicates that they mainly differ in the types of transcription factors and their binding sequences.

## Introduction

Flocculation is an inherent characteristic of yeasts involving asexual aggregation of cells into flocs that separate rapidly from the medium (reviewed recently in [1], [2]). Individual yeast cells transition into this morphological state as an adaptation to various environmental stresses by shielding the inner cells of the flocs [3]. The flocculent trait has also proven highly beneficial in industrial yeast applications by allowing efficient and cost-effective removal of cells [4]. The ability of yeast strains to flocculate is dependent on the expression of specific cell surface glycoproteins known as flocculins. Cell-to-cell adhesion occurs via binding between the flocculin and surface carbohydrates in a calcium-dependent manner [5]. The bound carbohydrates consist of various sugars including mannose, glucose, and galactose that are specific to the type of flocculin and yeast species [6]–[8]. There has been considerable interest in elucidating the genetic control of flocculation to better understand this phenomenon and generate biotechnological advances in yeast-based industries.

In *Saccharomyces cerevisiae*, a transcriptional-regulatory network composed of interactions between transcription factors and their flocculin gene targets is central in controlling flocculation. The primary flocculins that function in flocculation are encoded by the *FLO1*, *FLO5*, *FLO9*, and *FLO10* genes [9]–[11]. Overexpression of the individual *FLO* genes is sufficient to trigger flocculation [8], [12]. However, the degree of flocculation by *FLO* overexpression varies from *FLO1* to *FLO10* exhibiting the strongest to weakest flocculation, respectively. The flocculin *FLO11* also exhibits weak flocculation when overexpressed [8], but its function is mainly in cell-to-surface adhesion [13], diploid pseudohyphal growth [14], and haploid invasive growth [15]. The transcription factors required for flocculation include Flo8p and Mss11p, which primarily activate *FLO1* transcription [16]. The *Sacc. cerevisiae* laboratory strain *S288C* containing a nonfunctional *FLO8* gene is not able to flocculate, but flocculation is restored in this strain by the overexpression of *FLO8* or *MSS11* [16], [17]. In addition, Sfl1p has been shown to inhibit transcription of *FLO1* in the *W303-1A* strain and not in *S288C*, likely through interactions with the Ssn6p-Tup1p global repressor and components of Mediator [18], [19].

The control of flocculation is much less known in *Schizosaccharomyces pombe*. The ability of the heterothallic wild-type strains *972 h^−^* and *975 h^+^* to flocculate has not been observed presumably because the inducing environmental conditions have not been identified. Phenotypic analysis of constitutive flocculent mutant strains show that flocculation is dependent on the presence of calcium, but unlike *Sacc. cerevisiae*, the flocculin-carbohydrate interactions involve galactose rather than mannose and glucose residues [7]. Moreover, the transcriptional-regulatory network governing flocculation in *S. pombe* remains poorly characterized. Only a single interaction between the Mbx2 MADS box transcription factor and the *gsf2* ^+^ flocculin gene is currently known [20], [21]. The *gsf2^+^* gene was initially identified as highly upregulated in response to heterologous expression of *FLO8* [20]. Overexpression of *gsf2^+^* is sufficient to trigger flocculation while its deletion abrogates the flocculent phenotype of *tup12Δ*, *lkh1Δ*, and *gsf1* mutants. In addition, *gsf2^+^* displays additional roles in cell-to-surface adhesion and invasive growth [20]. The induction of *gsf2^+^* during flocculation and invasive growth is mediated by Mbx2 [21]. Two other transcription factors implicated in flocculation have been reported. The CSL transcription factors Cbf11 and Cbf12 play opposing roles in flocculation where mutant strains lacking *cbf11^+^* or overexpressing *cbf12^+^* flocculate [22]. The direct targets of these transcription factors functioning in flocculation have not been identified, but could be several putative flocculin genes that show protein sequence homology to other yeast-related proteins [23]. Indeed, these putative flocculin genes, as well as *gsf2* ^+^ are transcriptionally upregulated in certain Mediator mutants that flocculate indicating that these genes are likely repressed by Mediator [24]. Similar to *Sacc. cerevisiae*, the global transcriptional regulators Tup11 and Tup12 function in flocculation but their influence on the expression of these flocculin genes has not been addressed [25]. Importantly, it has not been directly demonstrated that these putative flocculin genes in *S. pombe* actually play a role in flocculation and the identity of the transcription factors that regulate them remains unknown.

In this study, we have initiated an extensive characterization of the transcriptional-regulatory network of *S. pombe* flocculation by identifying the relevant transcription factors and their flocculin gene targets. Importantly, we have also determined that heterothallic wild-type *S. pombe* is able to flocculate when grown in rich medium containing ethanol and glycerol as a carbon source. A screen of transcription factor deletion and overexpression strains for flocculent phenotypes revealed five novel transcriptional regulators of flocculation (Rfl1, Adn2, Adn3, Sre2, Yox1) in addition to our independent finding of Mbx2, Cbf11, and Cbf12. The strongest flocculation was observed upon overexpression of *mbx2^+^* and deletion of *rfl1^+^* (SPBC15D4.02) which encodes an uncharacterized fungal Zn(2)-Cys(6) transcription factor. Microarray expression profiling of the *mbx2OE* and *rfl1*Δ strains revealed good overlap in the upregulation of several flocculin genes, while ChIP-chip analysis of HA-tagged Mbx2 and Rfl1 under control of the *nmt41* promoter indicated that these transcription factors bound to some of the flocculin gene promoters. Nine flocculin gene targets (*pfl1* ^+^–*pfl9* ^+^) including *gsf2* ^+^/*pfl1^+^* were identified. The single overexpression of these genes triggered flocculation to varying degrees and cumulative effects on flocculation were observed in double overexpression experiments. Only loss of *gsf2^+^* could abrogate the flocculent phenotype of all the transcription factor mutants indicating that *gsf2* ^+^ encodes the dominant flocculin in *S. pombe*. Interestingly, we discovered that certain cell wall-remodeling enzymes can also function in flocculation, and some of these genes are likely regulated by the LisH transcription factors Adn2 and Adn3. In addition to the identification of target genes within the transcriptional-regulatory network, autoregulatory and inhibitory feed-forward loops involving several transcription factors were also detected. These results provide a significant insight into the transcriptional control of flocculation in *S. pombe*.

## Results

### Screening for novel transcription factors functioning in fission yeast flocculation

Our understanding of the transcriptional-regulatory network that governs flocculation in *S. pombe* remains limited. To further decipher this network, we sought to systematically identify transcription factors that play a role in flocculation. A list of 101 genes encoding sequence–specific transcription factors containing a bona-fide DNA-binding domain was assembled from [26] and GeneDB [27]. From this gene list, we constructed 101 *nmt1*-driven overexpression strains and 92 nonessential deletions in which the entire ORF was replaced with the KanMX6/NatMX6 cassette. A detailed description of the construction and phenotypic characterization of this transcription factor mutant collection will be described elsewhere (unpublished data). The transcription factor array of overexpression and deletion strains were screened for flocculation in EMM lacking thiamine and YES media, respectively. We recovered a total of eight transcription factors in which four overexpression strains (*mbx2OE*, *adn2OE*, *adn3OE* and *cbf12OE*) and four deletions (*rfl1*Δ, *sre2Δ*, *yox1Δ* and *cbf11Δ*) exhibited flocculation. These transcription factors represent positive and negative regulators of flocculation, respectively. Among these transcription factors, only the overexpression of *cbf12^+^* and *mbx2^+^* and deletion of *cbf11^+^* have been reported to cause flocculation [20], [22].

**Figure 1:**
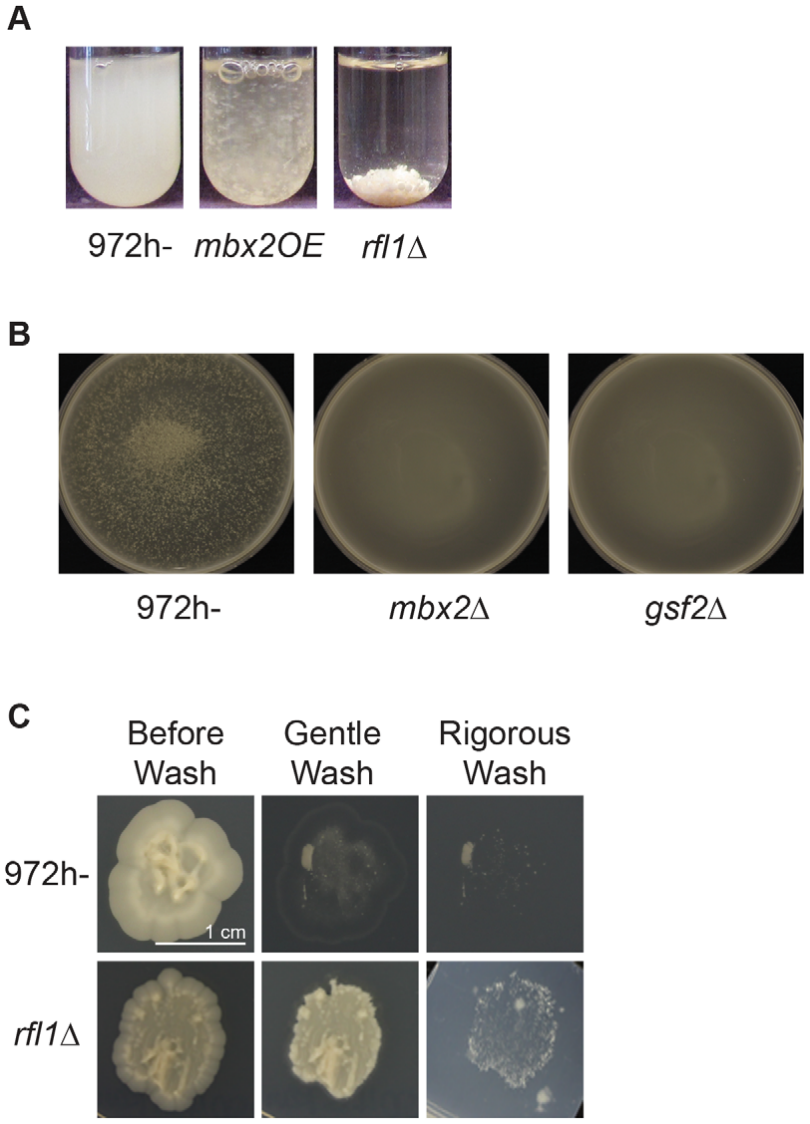
Flocculation induction by *rfl1^+^* deletion, *mbx2* ^+^ overexpression, or wild type grown in flocculation-inducing medium. (A) The flocculation of *rfl1*Δ mutant was visualized after culturing in YES medium for 24 hours at 30°C. The flocs of the *rfl1*Δ strain in YES medium were larger and sedimented faster than the flocs produced in the *nmt1*-driven *mbx2OE* strain after 48 hour induction. Due to fast settling of flocs, the culture tubes were shaken vigorously immediately prior to image capture. (B) Heterothallic wild-type cells (*972 h−*) flocculate when cultured in flocculation-inducing medium (1% yeast extract, 3% glycerol and 4% ethanol). However, deletion of *mbx2^+^* or *gsf2* ^+^ abolishes flocculation. Cells were inoculated in inducing medium at 10^6^ cells/ml and cultured for 5 days at 30°C, followed by petri dish assay (see materials and methods). (C) The *rfl1*Δ mutant exhibits enhanced adhesion to agar and invasive growth. Wild type (*972 h−*) and the *rfl1*Δ mutant were grown on LNB medium overlaid on YE+ALU medium without glucose for 10 days as per procedure outlined by Dodgson *et al.* [29]. Adhesion and invasive growth were determined by the amount of cells resistant to removal from the agar by gentle washing and more rigorous washing by rubbing cells off the agar with a finger under a stream of water, respectively.

The strongest flocculation was observed in the *mbx2OE* and *rfl1*Δ strains. The flocs of the *rfl1*Δ strain in YES medium were larger and sedimented faster than the flocs produced in the *mbx2OE* strain after 48 hour induction (Figure 1A). The *mbx2^+^* gene encodes a MADS-box transcription factor which was originally isolated in a screen for genes functioning in the biosynthesis of cell surface pyruvated galactose residues [28]. Recently, Mbx2 has been shown to function in flocculation and invasive growth by regulating the flocculin gene *gsf2^+^* [20], [21]. The *rfl1^+^* (*repressor of flocculation*) gene encodes an uncharacterized fungal Zn(2)-Cys(6) transcription factor.

The flocculation exhibited by these overexpression and deletion transcription factor mutants recovered from our screens could be abolished with the addition of galactose, but not mannose or glucose (data not shown). The amount of galactose required to completely deflocculate cells depended on the degree of flocculation. For example, *mbx2OE* strain could be deflocculated with 2% galactose while *rfl1*Δ strain required 5–10 times more galactose to completely deflocculate. Reflocculation of these strains was achieved in CaCl_2_ or in YES medium (data not shown).

The growth conditions that trigger flocculation in heterothallic wild-type *S. pombe* are not well known. To identify the inducing conditions, *972 h^−^* and *975 h^+^* cells were tested on different carbon sources at different cell densities for flocculation. We determined that heterothallic wild-type cells were able to flocculate when cultured for five days at an initial concentration of 1×10^6^ cells/ml in medium containing 1% yeast extract, 3% glycerol and, 4% ethanol (referred to as flocculation-inducing medium, Figure 1B). The degree of flocculation was slightly enhanced in strains auxotrophic for leucine, uracil, and/or adenine indicating that nutrient status may also play a role in triggering flocculation (data not shown). However, these wild-type strains flocculated significantly less in flocculation-inducing medium than the *mbx2OE* and *rfl1*Δ mutants in EMM and YES media, respectively. The weaker flocculation in these strains was more easily observed in petri-dishes incubated on an orbital rotator than in test tubes. In contrast to wild type, deletion of *mbx2^+^* did not produce any visible flocs in the flocculation-inducing medium (Figure 1B).

Fungal genes that function in flocculation are usually associated with filamentous invasive growth [17], [20]. We hypothesized that the *rfl1*Δ strain would exhibit hyperfilamentous invasive growth because of its strong flocculent phenotype. Indeed, the amount of cells resistant to removal from the agar by washing in the invasive assay on LNB medium with an underlayer of YE+ALU was much greater in the *rfl1*Δ strain than in wild type (Figure 1C). Under the microscope, the filamentous growth like those detected by Dodgson *et al.* [29] was observed below the agar surface for both wild type and *rfl1*Δ strain with the latter showing much larger and more frequent formation of filamentous growth (data not shown). Similarly, *adn2^+^* and *adn3^+^* which were previously observed to have defects in invasive growth when deleted were recovered in our screens as flocculent when overexpressed [29].

### Mbx2 and Rfl1 are opposing transcription factors that regulate putative flocculin genes

The strongest flocculation observed in the *mbx2OE* and *rfl1*Δ strains indicated that these two genes encode the major regulators of flocculation. Therefore, we initially focused on the characterization of these two transcription factors and proceeded to identify their target genes involved in flocculation. The *nmt41*-driven *mbx2-HA* strain was subjected to microarray expression profiling with a custom-designed *S. pombe* 8×15 K Agilent expression microarray (Table S2). The intermediate strength *nmt41* promoter was sufficient for *mbx2OE* flocculation and was utilized in the microarray experiments in order to reduce possible secondary transcriptional effects compared to the strong *nmt1* promoter. To better distinguish the direct target genes, ChIP-chip was also carried out concurrently on the same strain using the *S. pombe* 4×44 K Agilent Genome ChIP-on-chip microarray (Table S3). For the *rfl1* ^+^ expression profiling and ChIP-chip experiments, the flocculent deletion mutant and *nmt41*-driven *rfl1-HA* strain were used, respectively (Tables S4 and S5). The highly-induced putative target genes identified by microarray expression profiling of these transcription factor mutant strains were validated by qPCR (Table S13).

**Figure 2:**
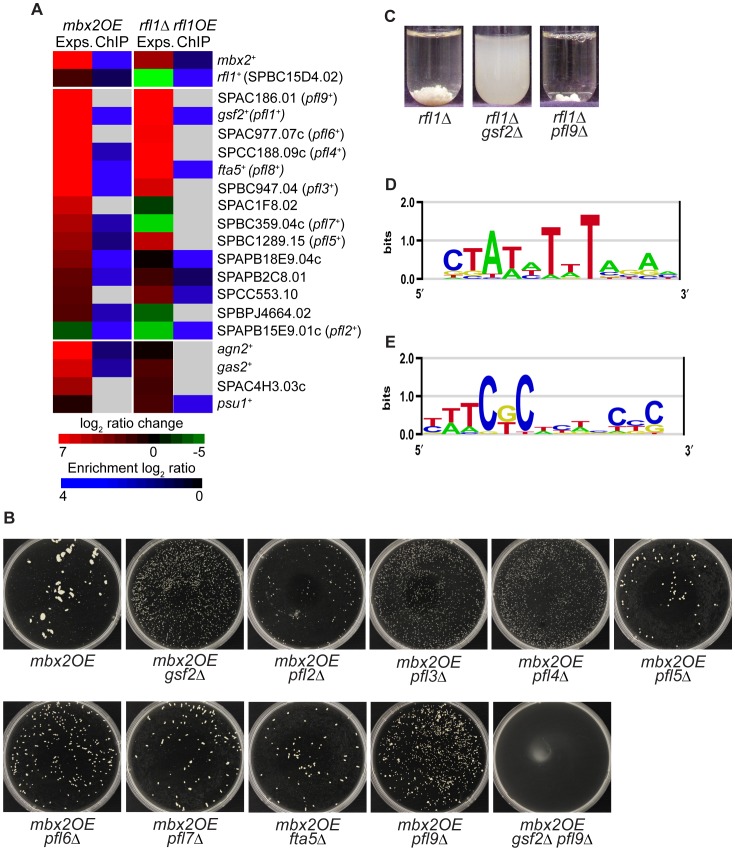
Transcriptional regulation of putative flocculin genes by Mbx2 and Rfl1. (A) The heat map shows the induction of several cell surface glycoprotein genes in *mbx2OE* and *rfl1*Δ strains and detection of their promoter occupancy by Mbx2 and Rfl1 (middle panel). Several genes encoding cell wall-remodeling enzymes were also induced in the *mbx2OE* strain, but not in the *rfl1*Δ strain (lower panel). Microarray expression profiling was performed with dye reversal on an *nmt41*-driven *mbx2-HA* strain and *rfl1*Δ mutant, while ChIP-chip analysis was carried out on *nmt41*-driven *mbx2-HA* and *rfl1-HA* strains. The color bars reflect relative expression and ChIP enrichment ratios between experimental and control strains. Light grey in the ChIP-chip clustergram indicates detection is below the threshold. (B) The deletion of putative flocculin target genes of Mbx2 and Rfl1 reduces the degree of flocculation as a result of *mbx2^+^* overexpression. An *nmt1*-driven *mbx2^+^* was overexpressed in single and double deletion strains of various putative flocculin genes in EMM minus thiamine medium for 24 hours and the degree of flocculation examined. (C) Deletion of *gsf2^+^* abrogates the flocculation of *rfl1*Δ cells. The mutant strains were grown to log phase in YES medium for 24 hours to assay flocculation. (D & E) Promoter analysis of differentially-expressed genes in *mbx2OE* (D) and *rfl1*Δ (E) strains. Putative DNA motifs were identified for Mbx2 and Rfl1 by RankMotif^++^.

The list of genes that were induced at least two fold in the *mbx2OE* or *rfl1*Δ strain was subjected to gene ontology analysis using the Princeton GO Term Finder (http://go.princeton.edu/cgi-bin/GOTermFinder). These induced genes were highly enriched in cell wall components with p-values of 9.0e-9 and 6.3e-6 for the *mbx2OE* and *rfl1*Δ strains, respectively. Strikingly, the most-induced genes in the *mbx2OE* strain encoded cell surface glycoproteins. The cell surface glycoprotein genes up-regulated above two-fold were SPAC186.01, *gsf2^+^*, SPAC977.07c/SPBC1348.08c, SPCC188.09c, *fta5^+^*, SPBC947.04, SPBC359.04c, SPBC1289.15, SPAPB2C8.01, SPAC1F8.02c, SPAPB18E9.04c, SPCC553.10, and SPBPJ4664.02, which all but *gsf2* ^+^ and the last 4 genes were predicted to be *pombe* adhesins based on BLAST sequence analysis (Figure 2A; [23]). SPAC977.07c and SPBC1348.08c are gene duplications with 100% sequence identity. To our knowledge, these genes with the exception of *gsf2^+^* have not been characterized further. The induction of these genes in the *mbx2OE* strain ranged from 2 to 112-fold relative to the empty vector control (Figure 2A, Table S13). In addition, several genes (*agn2^+^*, *psu1^+^*, SPAC4H3.03c and *gas2^+^*) encoding cell wall-remodeling enzymes such as glucan glucosidases and a betaglucanosyltransferase were induced up to 91-fold compared to the empty vector control when *mbx2^+^* was overexpressed (Figure 2A). In the *rfl1*Δ expression data, a similar set of cell surface glycoprotein genes were upregulated at a comparable level as the *mbx2OE* expression data except for SPAC1F8.02, SPBC359.04c, SPAPB18E9.04c and SPBPJ4664.02 (Figure 2A, Table S13). In contrast to the *mbx2OE* strain, the same genes encoding the cell wall-remodeling enzymes were not highly upregulated in the *rfl1*Δ strain (Figure 2A).

Of the thirteen highly-induced cell surface glycoprotein genes in the *mbx2OE* expression data, nine of them were detected with ChIP-chip indicating that these genes are very likely the direct transcriptional targets of Mbx2 (Figure 2A). Four of the nine highly-induced cell surface glycoprotein genes in the *rfl1*Δ strain were detected with ChIP-chip confirming that these genes are probably direct transcriptional targets of Rfl1 (Figure 2A). For both Mbx2 and Rfl1, *gsf2^+^*, *fta5* ^+^ and SPAPB2C8.01 were detected in the expression microarray and ChIP-chip experiments (Figure 2A).

Next, we sought further evidence that these cell surface glycoprotein genes were targets of Mbx2 and Rfl1 by epistasis studies. We decided to study a subset of these genes, which included the majority of the gene sequences analyzed by Linder and Gustafsson [23], [24]. The *mbx2* ^+^ gene was overexpressed in single deletions of these putative target genes and their degree of flocculation was determined visually in petri-dishes, as well as quantitatively (Table S14). The putative glycoprotein gene SPAPB15E9.01c was included in these studies, because even though the transcript was downregulated in both *mbx2OE* and *rfl1*Δ strains, ChIP-chip analysis detected Mbx2 and Rfl1 association with its promoter (Figure 2A). Deletion of *gsf2^+^* decreased *mbx2OE* flocculation to the greatest extent while the reduction of flocculation was less extensive in the other single deletion mutants (Figure 2B, Table S14). The degree of reduction in *mbx2OE* flocculation roughly corresponded to the *pfl* numbers, which were assigned based on the degree of flocculation when overexpressed (see below). Moreover, *mbx2OE* flocculation was completely abrogated in the *gsf2Δ pfl9*Δ double mutant indicating that the reduction of *mbx2OE* flocculation in these mutants were additive in some cases (Figure 2B). Similar experiments were performed for *rfl1^+^* in which flocculation was assayed in the same putative target deletions in the *rfl1*Δ background. The flocculation exhibited in the *rfl1*Δ strain was completely abolished by the deletion of *gsf2^+^*, but not by the deletion of *pfl9* ^+^ (Figure 2C).

To further analyze the expression microarray datasets of Mbx2 and Rfl1, the promoter regions of the differentially-expressed genes were subjected to the motif-finding algorithms RankMotif^++^ and MEME to identify their binding specificities [30], [31]. Mbx2 is a member of the MEF2-MADS box transcription factor family which has been shown to bind to the consensus sequence 5′-(C/T)TA(T/A)_4_TA(G/A)-3′ [28], [32], [33]. The Mbx2 binding specificity obtained by RankMotif^++^ closely resembled this known consensus sequence (Figure 2D). Similarly, RankMotif^++^ generated an Rfl1 binding specificity that resembled known consensus sequences of several members of the fungal Zn(2)-Cys(6) transcription factor family (Figure 2E). The binding specificity of Zn(2)-Cys(6) DNA-binding domains is composed of conserved GC-rich trinucleotides spaced by a variable sequence region differing in length among members of the transcription factor family [34]. Analyses of the Mbx2 and Rfl1 expression microarray and ChIP-chip datasets by MEME did not generate any candidate DNA motifs.

Altogether, these results demonstrate that Mbx2 and Rfl1 are transcription factors responsible for regulation of flocculation in fission yeast by activating or repressing the transcription of candidate *S. pombe* flocculin genes, respectively.

### The putative flocculin gene targets of Mbx2 and Rfl1 are sufficient to induce flocculation when overexpressed

**Figure 3:**
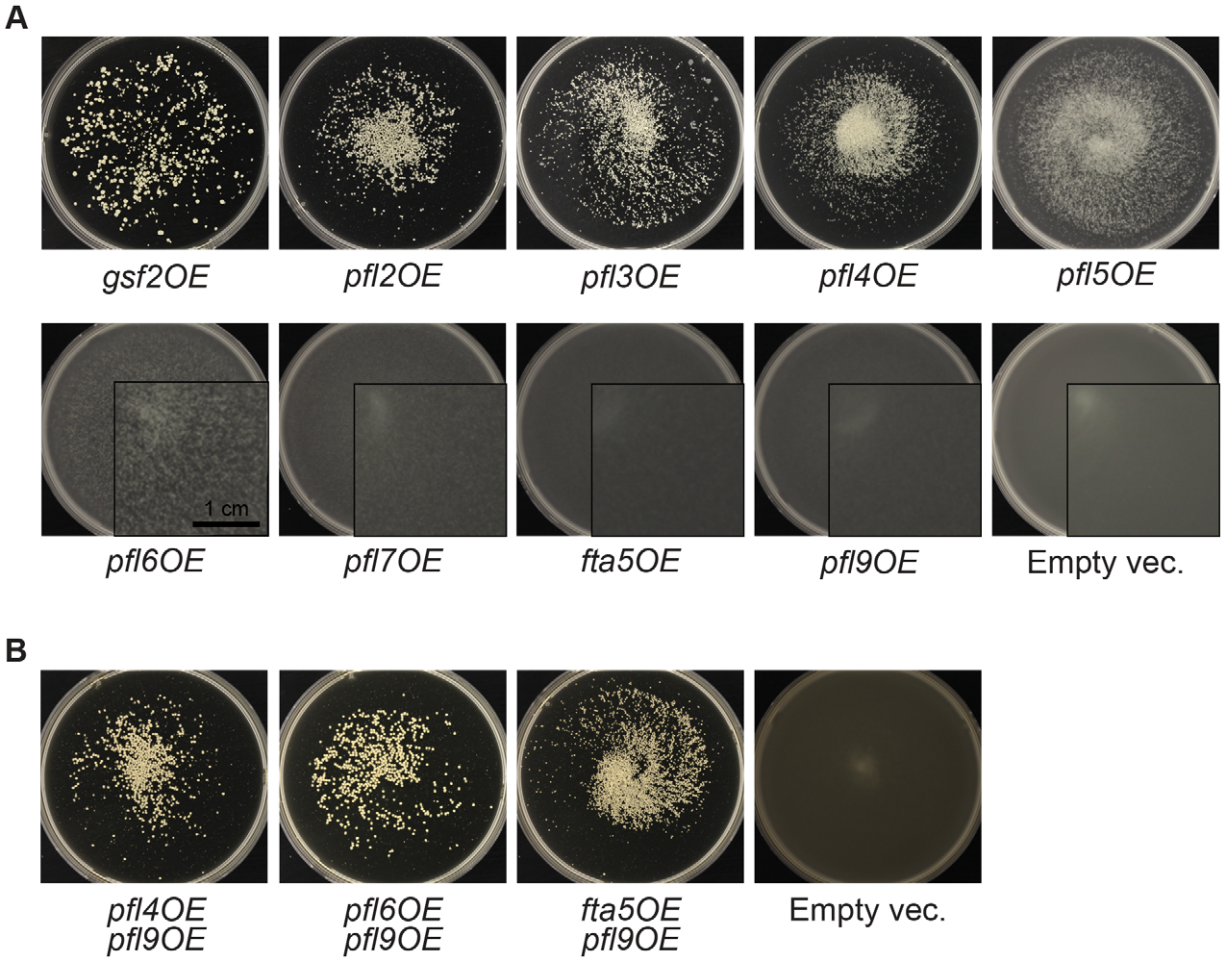
Overexpression of putative flocculin target genes of Mbx2 and Rfl1 induces flocculation. (A) Single overexpression of the Mbx2 and Rfl1 flocculin target genes (*gsf2^+^*/*pfl1^+^* and *pfl2^+^*–*pfl9^+^*) with the *nmt1* promoter induces flocculation to varying degrees. The number assigned to the *pfl^+^* genes corresponds roughly to the relative strength of flocculation upon overexpression (i.e. *gsf2^+^*/*pfl1^+^* to *pfl9^+^* showing strongest to mildest flocculation, respectively). The overexpression strains were cultured for total of 7 days (sub-cultured into fresh medium on third day) in EMM minus thiamine medium at 30°C. (B) The flocculin genes exhibit additive effects on flocculation. Double overexpression of various flocculin genes resulted in greater flocculation than the overexpression of the single corresponding genes.

Besides *gsf2^+^*, the other putative target genes of Mbx2 and Rfl1 that encode for cell surface glycoproteins share some amino acid sequence homology with domains found in other fungal adhesins [23]. However, the role of these glycoprotein genes in flocculation has not been demonstrated. Overexpression studies were employed to the aforementioned set of putative flocculin target genes of Mbx2 and Rfl1 to determine whether they function directly in flocculation. Each single overexpression of these flocculin genes was able to induce flocculation to varying degrees with the strongest flocculation observed in the *gsf2OE* strain which produced visible flocs within one day (Figure 3A; Table S14). Weaker flocculation was observed from the overexpression of the other flocculin genes after total incubation of 2–7 days in EMM minus thiamine medium with sub-culturing into fresh medium in Day 3. The flocculation images of these overexpression strains shown in Figure 3A were captured after total of 7 days of induction. As a result of these observations, we named these genes *pfl^+^* for *Pombe Flocculins* and numbered them according to their degree of flocculation when overexpressed: *pfl1^+^*/*gsf2^+^* (referred as *gsf2^+^* hereafter), *pfl2^+^*/SPAPB15E9.01c, *pfl3^+^*/SPBC947.04, *pfl4^+^*/SPCC188.09c, *pfl5^+^*/SPBC1289.15, *pfl6^+^*/SPAC977.07c, *pfl7^+^*/SPBC359.04c, *pfl8^+^*/*fta5^+^* (referred as *fta5* ^+^ hereafter) and *pfl9^+^*/SPAC186.01. Furthermore, we overexpressed some double combinations of the weaker flocculin genes to determine whether flocculation could be additive. Indeed, the *pfl4^+^ pfl9^+^*, *pfl6^+^ pfl9^+^*, and *fta5^+^ pfl9^+^* double overexpression strains flocculated earlier and formed larger flocs than their corresponding single overexpressors, thus, demonstrating the additive effect of these flocculins (Figure 3B, Table S14). We next tested the single deletions of the *pfl^+^* genes for their ability to flocculate in flocculation-inducing medium. No visible flocculation was observed in the *gsf2*Δ strain while wild type was flocculent (Figure 1B). In contrast, flocculation still occurred in the *pfl2Δ*–*pfl9Δ* strains in the inducing medium indicating that *gsf2^+^* encodes the dominant flocculin and the other flocculin genes are dispensable for flocculation (data not shown).

These observations revealed that the contribution in flocculation by these *pfl^+^* genes varied and certain combinations of *pfl^+^* were additive. The strength of flocculation by the single overexpression of *pfl^+^* genes was directly correlated with the reduction of *mbx2OE* flocculation in the corresponding deletion strains (Figure 2B and Figure 3A, Table S14). For example, the *pfl2OE* strain which produced larger flocs than the *pfl3OE*–*pfl9OE* strains exhibited a greater inhibition of *mbx2OE* flocculation when deleted. Similarly, the flocculation of the *rfl1*Δ strain was completely abrogated by the deletion of *gsf2* ^+^, but not at all by the deletion of *pfl9* ^+^ (Figure 2C). Consistent with the above results, the deletion of both *gsf2* ^+^ and *pfl9* ^+^ led to a greater abrogation of *mbx2OE* flocculation compared to each deletion alone (Figure 2B). In summary, we have demonstrated that these *pfl^+^* genes encode for *S. pombe* flocculins and Gsf2 is the dominant flocculin.

### Positive and negative autoregulation of *mbx2^+^* and *rfl1^+^*, respectively

**Figure 4:**
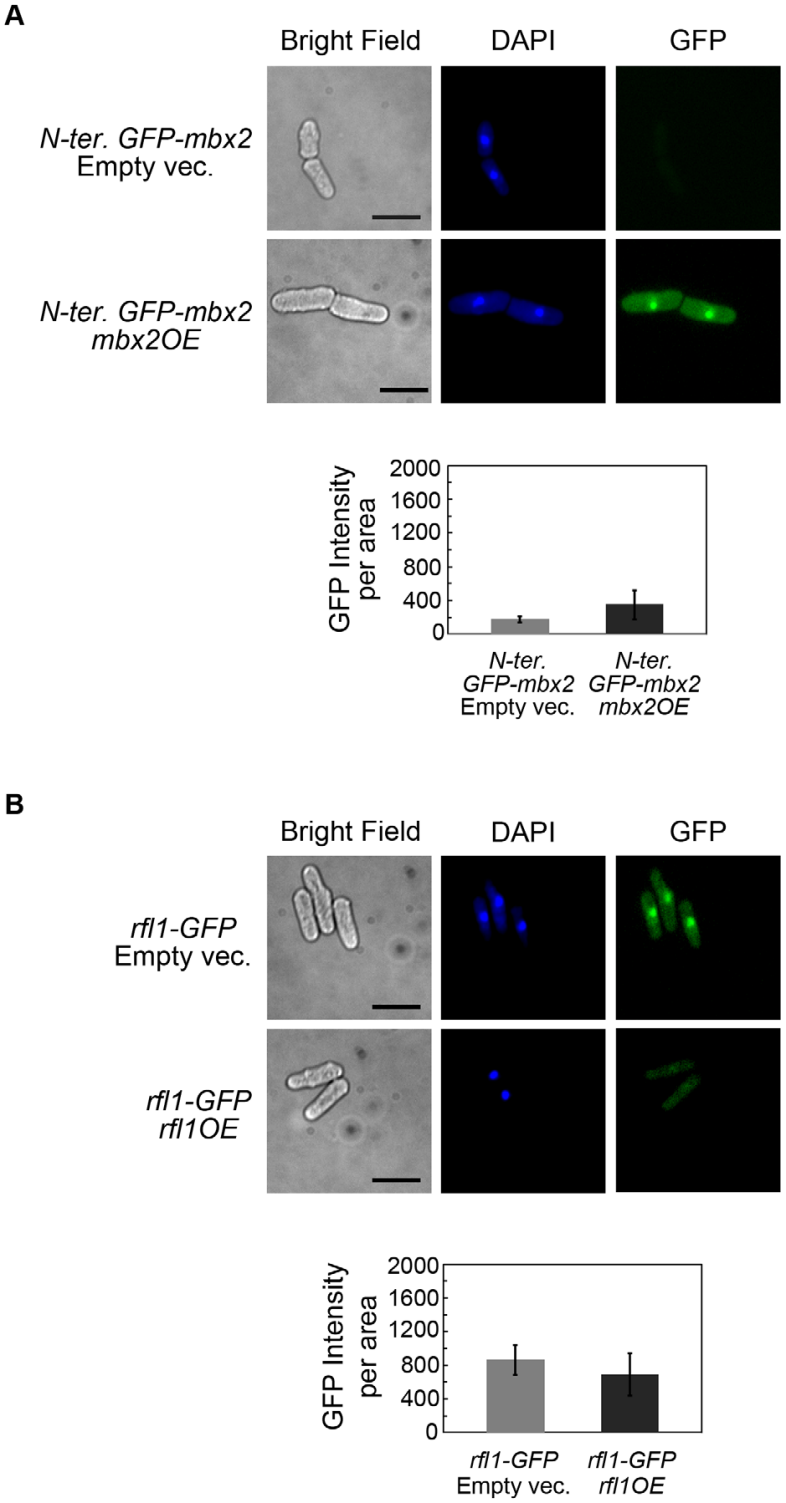
*mbx2^+^* and *rfl1^+^* undergo positive and negative autoregulation, respectively. (A) Positive autoregulation of *mbx2^+^*. A strain containing N-terminal GFP-tagged *mbx2^+^* under the control of its native promoter displayed increased GFP expression when *mbx2^+^* was ectopically expressed with the *nmt1* promoter. Nuclear GFP-Mbx2 signal and flocs in liquid culture were detected at the 9 hour induction of *nmt1*-driven *mbx2^+^* in EMM minus thiamine medium. Cells were deflocculated in 2% galactose prior to fluorescence microscopy to facilitate image acquisition. The presence of galactose does not affect the GFP signal (data not shown). The bar graph compares the mean and standard deviation of cellular GFP-Mbx2 signal resulting from *nmt1*-driven *mbx2^+^* and empty vector control with a significant difference of p<0.001 (Welch's two tailed *t*-test; n = 50, df = 52). (B) Negative autoregulation of *rfl1^+^*. A strain containing C-terminal GFP-tagged *rfl1^+^* under native control exhibited nuclear expression (empty vector). Ectopic expression of *nmt1*-driven *rfl1^+^* for 18 hours in EMM minus thiamine medium reduced the nuclear GFP signal with a slight increase in cytoplasmic GFP signal. The bar graph compares the mean and standard deviation of overall cellular Rfl1-GFP signal resulting from *nmt1*-driven *rfl1^+^* and empty vector control with a significant difference of p<0.01 (Welch's two tailed *t*-test; n = 27, df = 44). Cells were stained with DAPI to visualize nuclei. Scale bar, 10 µm.

Interestingly, ChIP-chip analysis also detected binding of Mbx2 and Rfl1 to their own promoters, as well as Rfl1 binding to the *mbx2* ^+^ promoter (Figure 2A), indicating autoregulation and *mbx2^+^* regulation by Rfl1 within the transcriptional-regulatory network of *S. pombe* flocculation. Mbx2 also appeared to be associated with the *rfl1^+^* promoter, but this interaction was marginal as it was found just above the detection threshold for ChIP-chip (Figure 2A). To investigate the autoregulation of *mbx2* ^+^, the gene was C-terminal tagged with GFP at its native locus (*mbx2*-*GFP*). However, the GFP-tagged strain resulted in a hypermorphic allele that displayed constitutive flocculation and nuclear localization of Mbx2-GFP (see below). We speculated that the removal of the 3′-untranslated region of *mbx2* ^+^ during the C-terminal tagging may be the cause of the hypermorphic allele. To bypass this potential problem, we created an N-terminal GFP-tagged allele (*GFP*-*mbx2*) with an intact 5′-untranslated region and approximately 1 kb of native promoter sequence. In contrast to the C-terminal tagged hypermorphic allele, the N-terminal tagged GFP-Mbx2 expression was comparable to background levels and the strain did not exhibit constitutive flocculation (Figure 4A). Moreover, the *GFP-mbx2* strain flocculated when grown in glycerol-inducing medium indicating that the tagged protein is functional (Table S14). When *nmt1*-driven *mbx2* ^+^ expression was induced for 9 hours in the *GFP*-*mbx2* strain, nuclear GFP-Mbx2 expression was detected, indicating that Mbx2 can activate its own expression (Figure 4A). As expected, this strain was now flocculent. Longer induction of *nmt1*-driven *mbx2* ^+^ expression resulted in greater GFP-Mbx2 expression with multi-nucleated GFP foci (data not shown). The positive autoregulation of *mbx2* ^+^ is likely to be direct as several putative MEF2-binding sequences (e.g. 5′-TTAAAAATAG-3′) are located within 1000 bp upstream from the *mbx2* ^+^ start codon (data not shown).

To determine whether negative autoregulation occurs with *rfl1* ^+^, a C-terminal GFP-tagged strain under native control was generated (*rfl1*-*GFP*). The localization of Rfl1-GFP was nuclear in the *rfl1*-*GFP* strain (Figure 4B). The induction of *nmt1*-driven *rfl1* ^+^ expression for 18 hours in the *rfl1*-*GFP* strain led to a reduced nuclear Rfl1-GFP signal and a slightly increased cytoplasmic Rfl1-GFP signal (Figure 4B). However, overall Rfl1-GFP expression in the cell was reduced when Rfl1 was overexpressed compared to the empty vector control (Figure 4B; two-tailed *t*-test; p value<0.01). In contrast to our observations with the Rfl1-GFP protein expression, we found that there was no decrease of the Rfl1-GFP transcript when *rfl1^+^* was overexpressed (Table S13). These results indicate that although Rfl1 can bind to its own promoter, negative autoregulation appears marginal or may not be occurring.

### Rfl1 represses *mbx2^+^* expression

**Figure 5:**
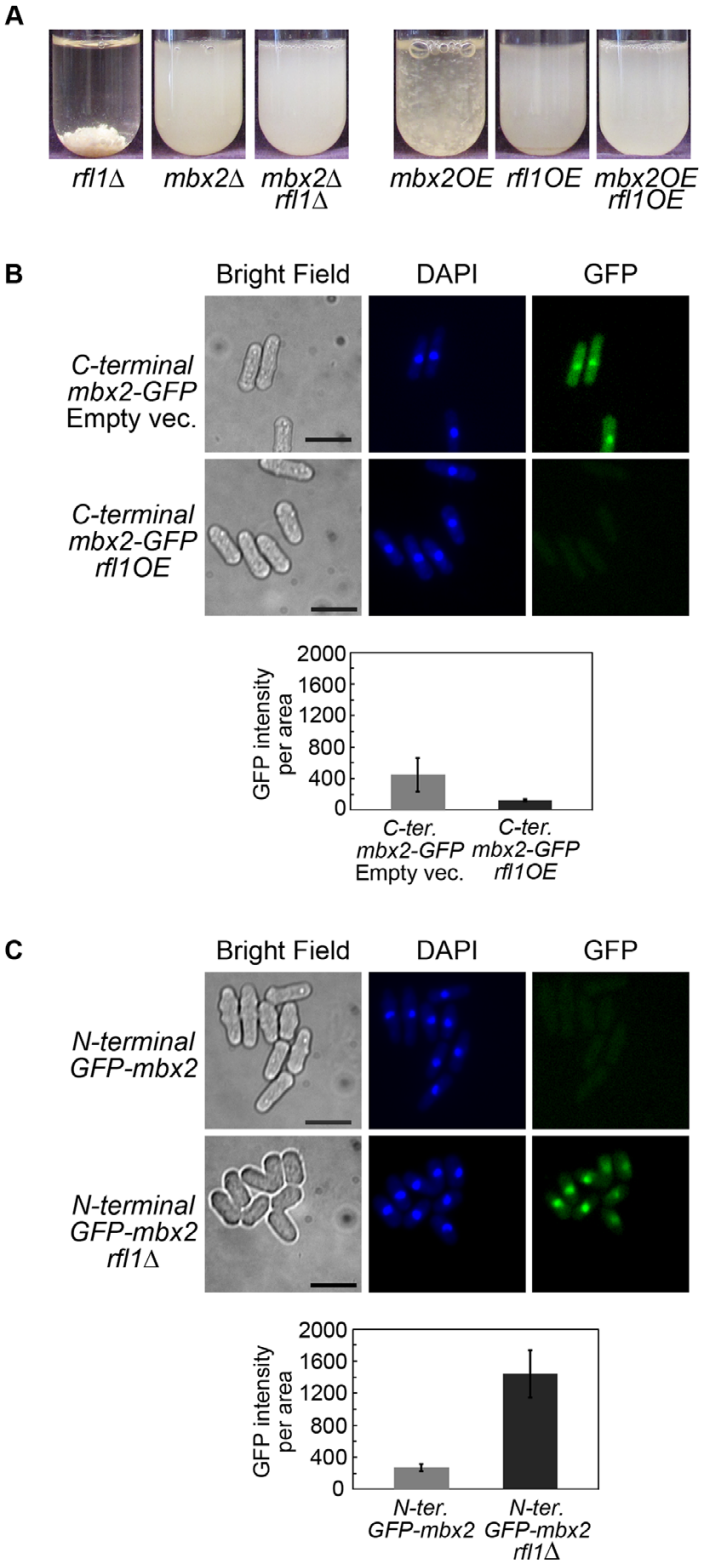
Rfl1 represses *mbx2^+^* expression. (A) Genetic interactions between *mbx2^+^* and *rfl1^+^*. The flocculation phenotype of the *rfl1*Δ mutant is abolished by deletion of *mbx2^+^* (left panel). In addition, the flocculation caused by overexpression of *mbx2^+^* is abolished by *rfl1^+^* overexpression (right panel). The deletion and overexpression strains were cultured in YES and EMM minus thiamine media, respectively, for 24 hours at 30°C. (B) The expression of C-terminal GFP-tagged *mbx2^+^* under native control is reduced by ectopic expression of *rfl1^+^*. The C-terminal GFP-tagged *mbx2^+^* strain is a hypermorphic allele that exhibits constitutive flocculation and nuclear Mbx2-GFP expression. The C-terminal GFP-tagged *mbx2^+^* strain containing *nmt1*-driven *rfl1^+^* or empty vector was inoculated at approximately 10^4^ cells/ml in EMM minus thiamine medium and cultured for 20–30 hours at 30°C until late log phase. The strain containing the empty vector was deflocculated in 2% galactose prior to fluorescence microscopy. This procedure was not required for the *rfl1OE* strain because it was no longer flocculent. (C) Deletion of *rfl1^+^* results in the expression of native-controlled N-terminal GFP-tagged *mbx2^+^*. The N-terminal GFP-tagged *mbx2^+^* strain in a wild type and *rfl1*Δ background were cultured to mid-log phase in YES medium. The bar graph compares the mean and standard deviation of overall cellular Mbx2-GFP (B) and GFP-Mbx2 (C) signals between the experimental and control strains with significant difference of p<0.001 (Welch's two tailed *t*-test; n = 50, df = 50 for each experiment). Cells were stained with DAPI to visualize nuclei. Scale bar, 10 µm.

The observation that Rfl1 is associated with the *mbx2* ^+^ promoter by ChIP-chip suggests that Rfl1 may oppose Mbx2 function in flocculation by repressing its expression. To test this hypothesis, we first examined the genetic interactions between *mbx2^+^* and *rfl1^+^*. The *mbx2Δ rfl1Δ* double mutant did not display flocculation indicating that *mbx2^+^* is epistatic to *rfl1^+^* (Figure 5A). In addition, the flocculation associated with *mbx2OE* was abrogated by co-overexpression of *rfl1^+^* (Figure 5A). These results are consistent with *mbx2* ^+^ being downstream of *rfl1* ^+^ and that *rfl1* ^+^ opposes *mbx2* ^+^ function in flocculation.

We next utilized the C-terminal and N-terminal GFP-tagged *mbx2* ^+^ strains to further determine if Rfl1 represses *mbx2^+^* expression. First, Rfl1 was overexpressed in the hypermorphic C-terminal tagged *mbx2*-*GFP* allele which shows constitutive nuclear Mbx2-GFP expression and flocculation. This resulted in the near-abolishment of both the GFP signal (Figure 5B) and flocculation (data not shown) in the hypermorphic *mbx2* allele. Second, when the N-terminal tagged *GFP*-*mbx2* strain was crossed into the *rfl1*Δ background, the resulting strain displayed dramatic increase in nuclear GFP-Mbx2 expression (Figure 5C) and flocculation strength equivalent to the *rfl1*Δ strain (data not shown). These results support the hypothesis that *mbx2^+^* expression is repressed by Rfl1 in non-flocculent cells.

### Overexpression of *cbf12^+^* causes flocculation due to up-regulation of *gsf2^+^*

Cbf12, a member of the CSL transcription factor family has previously been reported to trigger flocculation when overexpressed [22]. However, the target genes of Cbf12 that function in flocculation have not been identified. To further elucidate the role of *cbf12* ^+^ in flocculation, we took a similar approach to identify its direct target genes by concurrent expression microarray profiling and ChIP-chip analysis of the *nmt41*-driven *cbf12-HA* strain (Tables S6 and S7, respectively).

**Figure 6:**
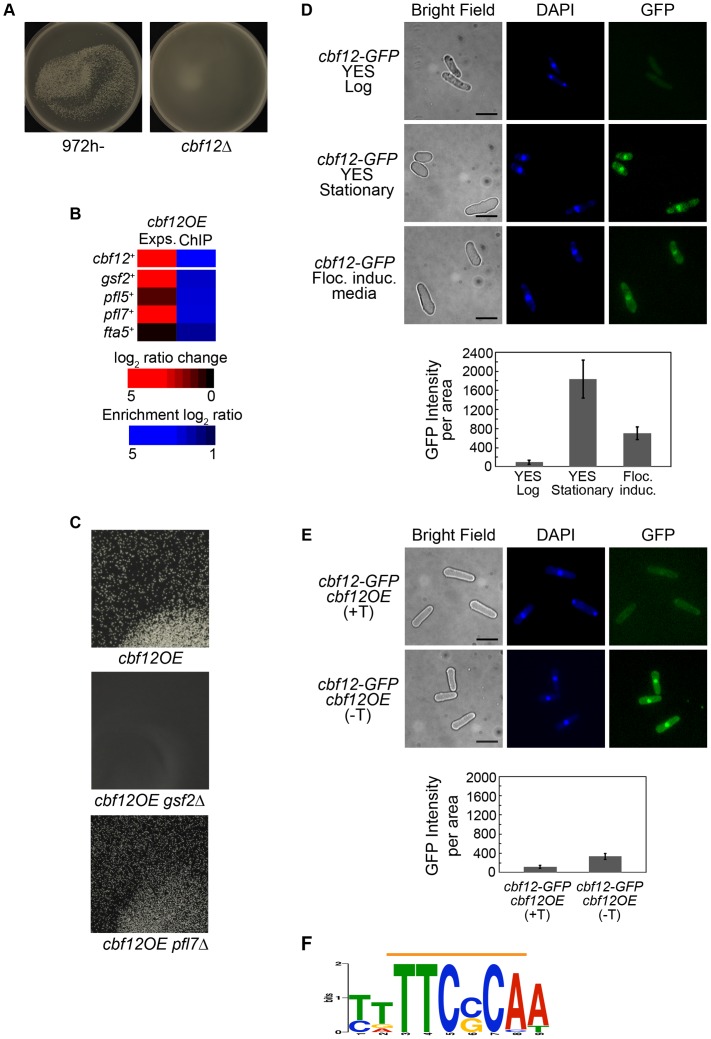
Regulation of flocculation by Cbf12. (A) Loss of *cbf12^+^* prevents flocculation under inducing conditions. Wild type and the *cbf12Δ* mutant were cultured in flocculation-inducing medium for 5 days at 30°C. (B) Cbf12 regulates putative flocculin genes. The heat map shows induction of several flocculin genes and their promoter occupancy by Cbf12 from microarray expression profiling and ChIP-chip analysis, respectively, of an *nmt41*-driven *cbf12-HA* strain. The color bars reflect relative expression and ChIP enrichment ratios between experimental and control. (C) The absence of *gsf2^+^*, but not *pfl7^+^* abolishes the flocculation triggered by *cbf12^+^* overexpression. Strains containing *nmt1*-driven *cbf12^+^* in wild type, *gsf2*Δ or *pfl7Δ* backgrounds were cultured for 24 hours in EMM minus thiamine medium at 30°C. Approximately 1/16 of the petri-dish was magnified to reveal more details of the flocs. (D) *cbf12^+^* is expressed in wild-type cells grown in rich medium at stationary phase and in flocculation-inducing medium, but not in rich medium at log phase. A C-terminal GFP-tagged *cbf12^+^* strain under the control of its native promoter was grown to log or stationary phase in YES or in the inducing medium at 30°C. The bar graph compares the mean and standard deviation of cellular Cbf12-GFP signals. (E) Positive autoregulation of *cbf12^+^*. Ectopic expression of *nmt1*-driven *cbf12^+^* results in the upregulation of native-controlled Cbf12-GFP expression in log-phase cells. The bar graph compares the mean and standard deviation of cellular Cbf12-GFP signals between induced and uninduced *cbf12OE* cells with significant difference of p<0.001 (Welch's two tailed *t*-test; n = 19, df = 25). (F) A DNA motif closely matching the binding specificity of CSL transcription factors was retrieved from the *cbf12OE* microarray expression data. The promoter region (1000 base pairs upstream of the start codon) of 11 highly-induced genes encoding for cell surface proteins as identified by the Princeton GO Term Finder was applied to MEME using default settings. The orange line indicates bases that match with the known binding site of CSL transcription factors. Cells were stained with DAPI to visualize nuclei. Scale bar, 10 µm.

When *cbf12* ^+^ was deleted and cultured in flocculation-inducing medium, flocculation was abolished (Figure 6A). In contrast, overexpression of *cbf12* ^+^ by the *nmt1* promoter triggered flocculation (Figure 6C) and produced a bowling pin–shaped phenotype after 24 hours in medium lacking thiamine (data not shown). Further induction of the *nmt1*-driven *cbf12* ^+^ caused the strain to become sick and granulated, eventually leading to growth arrest (data not shown). To reduce the toxic effects of *cbf12^+^* overexpression, an *nmt41*-driven *cbf12-HA* strain was used for concurrent expression profiling and ChIP-chip analysis.

Gene ontology analysis was carried out separately on the top 50 most highly-induced genes and all 160 promoter-occupied genes by Cbf12 with the Princeton GO Term Finder. Functional enrichment of genes in cell surface (p = 1.8e-7) and plasma membrane (p = 5.7e-4) was detected for the highly-induced and promoter-occupied genes, respectively. These genes included several flocculin genes, (Figure 6B). Both *gsf2* ^+^ and *pfl7* ^+^ were among the five highest induced genes (18.1 and 27.6-fold, respectively) in the *cbf12OE* strain and were also detected by ChIP-chip (Figure 6B) suggesting that Cbf12 directly activates the transcription of *gsf2* ^+^ and *pfl7* ^+^ for flocculation. The flocculation triggered by *cbf12^+^* overexpression was completely abrogated in the *gsf2*Δ background, whereas deletion of *pfl7^+^* had little effect (Figure 6C, Table S14). This was consistent with the hypothesis that *gsf2* ^+^ encodes the dominant flocculin. In addition, loss of *gsf2* ^+^ or *pfl7* ^+^ did not alter the bowling-pin cell shape or the reduced fitness phenotypes of the *cbf12OE* strain indicating that these two phenotypes were not due to the upregulation of the flocculin genes (data not shown). The much weaker flocculation observed in the *cbf12OE* strain in comparison to the *mbx2OE* and *gsf2OE* strains may be attributed to additional defects in cell and nuclear division, which would cause early growth arrest before the full flocculation potential could be reached [22].

Consistent with previous findings, C-terminal GFP-tagged Cbf12 under native control was expressed predominantly in the nucleus in stationary phase cells while expression in logarithmic cells was comparable to background (Figure 6D; [22]). Compared to logarithmic growth in rich medium, Cbf12-GFP nuclear expression increased in cells grown in flocculation-inducing medium, thus supporting its role in flocculation (Figure 6D). Interestingly, Cbf12 was also detected by ChIP-chip to bind to its own promoter (Figure 6B). Indeed, positive autoregulation appears to occur as native Cbf12-GFP expression increased greater than three-fold when *nmt1*-driven *cbf12* ^+^ was ectopically expressed in logarithmically growing cells (Figure 6E).

Recently, it was demonstrated that an N-terminal-truncated Cbf12 bound to probes containing a canonical CSL binding motif (5′-GTGGGAA-3′) by gel mobility shift assay [35]. We next searched for a similar DNA binding sequence for Cbf12 from the expression microarray and ChIP-chip *cbf12OE* datasets by RankMotif^++^ and MEME. RankMotif^++^ and MEME analyses of the expression microarray and ChIP-chip data, respectively, did not identify a binding specificity for Cbf12. However, when the promoters of up-regulated genes in the *cbf12OE* strain belonging to the cell surface GO category were subjected to MEME analysis, a motif closely matching the canonical CSL binding motif (6/7 nucleotide match) was recovered (Figure 6F).

These results demonstrate Cbf12 as part of the transcriptional-regulatory network of fission yeast flocculation by controlling the transcription of several flocculin genes including *gsf2^+^*.

### The flocculation function of *yox1^+^*, *sre2^+^*, and *cbf11^+^* is dependent on *gsf2^+^*

**Figure 7:**
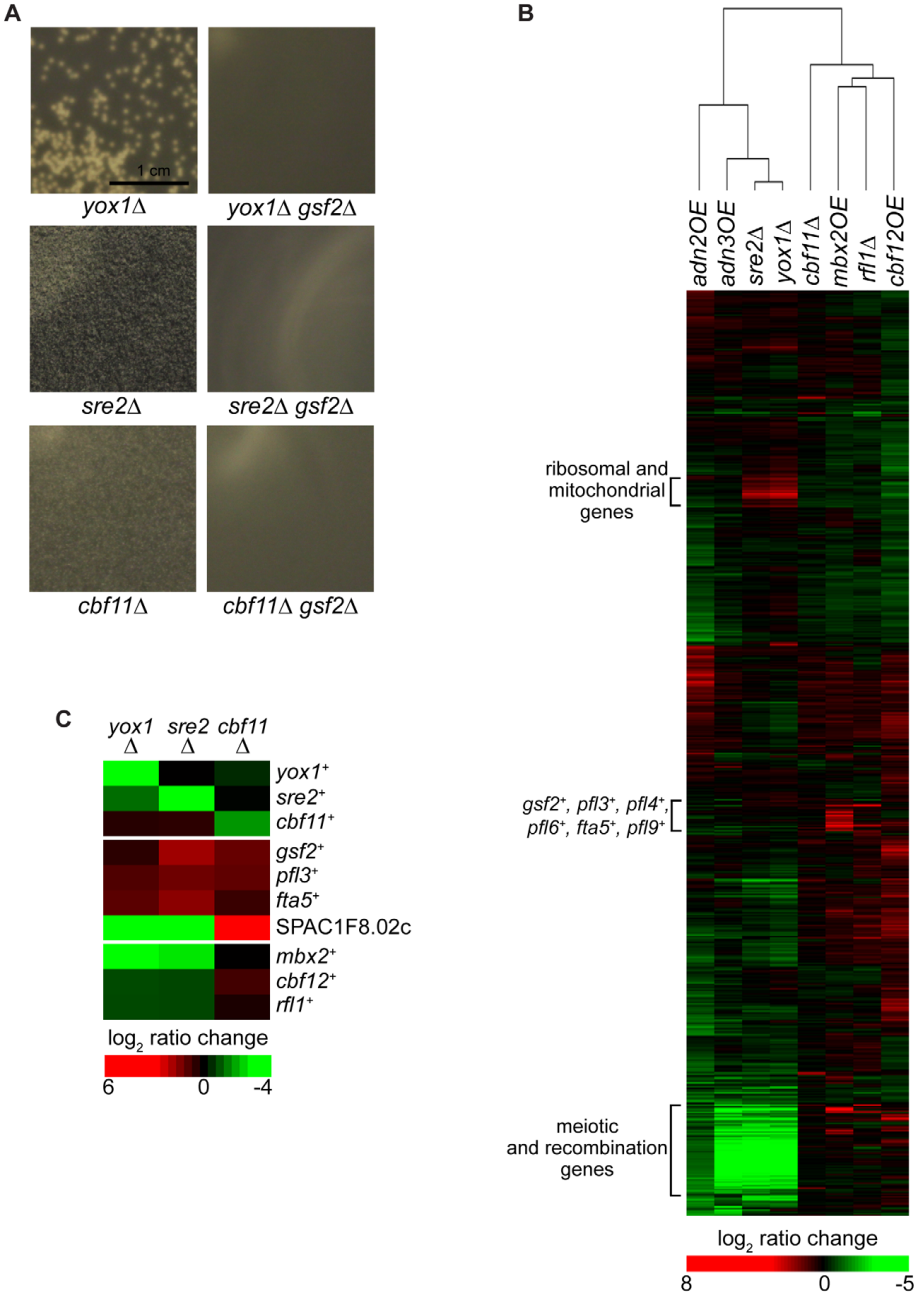
Regulation of flocculation by Yox1, Sre2, and Cbf11. (A) The regulation of flocculation by Yox1, Sre2 and Cbf11 is dependent on *gsf2^+^*. *yox1Δ*, *sre2Δ* and *cbf11Δ* cells flocculate in late log and stationary phase when grown in YES medium (left panels). The flocculation of these mutant strains was abrogated in the *gsf2*Δ background (right panels). (B) Clustergram of microarray expression profiles of flocculent transcription factor mutants. The microarray expression profiles of the *yox1Δ* and *sre2Δ* strains are most similar and show upregulation of ribosomal and mitochondrial genes. (C) The *yox1Δ*, *sre2Δ* and *cbf11Δ* flocculent strains show upregulation of several flocculin genes. The color bars reflect relative expression between experimental and control.

From our transcription factor screens, the deletion of *yox1^+^*, *sre2^+^*, or *cbf11^+^* also resulted in flocculation, although the size of the flocs were smaller than observed in *mbx2OE*, *cbf12OE* and *rfl1*Δ strains (Figure 7A, Table S14). Yox1 has been implicated in a negative autoregulatory loop to prevent inappropriate transcriptional expression of MBF gene targets, while the function of Sre2, which shows homology to the human sterol regulatory element binding protein SREBP-1A remains largely unknown [36], [37]. A role of Yox1 and Sre2 in flocculation has not been reported. In contrast, *cbf11* ^+^ encodes a CSL transcription factor that plays a role in flocculation, but its target genes are not known [22].

To elucidate the transcriptional flocculation program of *yox1^+^*, *sre2^+^* and *cbf11^+^*, expression microarray profiling was conducted on the corresponding flocculent deletion strains in rich medium (Tables S8, S9, S10). The expression microarray profiles of *yox1*Δ and *sre2Δ* most resembled each other compared to the other strains described in this study (Figure 7B). Genes upregulated by at least two-fold in the *yox1Δ* and *sre2Δ* strains showed enrichment for ribosomal subunits (p = 2.8e-31 and 7.4e-25 for *yox1Δ* and *sre2Δ*, respectively) and mitochondrial membrane transporters (p = 7.5e-5 and 1.2e-3 for *yox1Δ* and *sre2Δ*, respectively). These findings did not intuitively answer our questions as to how these two transcription factors might be related or associated with the flocculation pathway. We next examined whether any of the flocculin genes and their putative regulators were induced in the *yox1Δ* and *sre2Δ* strains. In the *sre2Δ* strain, *gsf2* ^+^, *pfl3* ^+^ and *fta5* ^+^ transcripts were upregulated 3.7, 2.5 and 3.1-fold, respectively, indicating that the expression of these genes could be contributing to the flocculent phenotype (Figure 7C). In contrast, *mbx2* ^+^ and *cbf12* ^+^ transcripts were downregulated approximately 2-fold suggesting that the elevated levels of *gsf2* ^+^, *pfl3* ^+^ and *fta5* ^+^ transcripts in the *sre2Δ* strain were not mediated by Mbx2 and Cbf12 (Figure 7C). Similarly in the *yox1Δ* strain, we observed that *gsf2^+^* and *pfl3* ^+^ transcripts were upregulated although less than in the *sre2Δ* strain, and *mbx2* ^+^ and *cbf12* ^+^ were also downregulated (Figure 7C). Therefore, this suggests that *sre2* ^+^ and *yox1* ^+^ may be involved in the repression of flocculation through a pathway independent from *mbx2* ^+^ and *cbf12* ^+^.

The microarray expression profile of the *cbf11Δ* strain revealed greater than 2-fold increase of *gsf2^+^* and *pfl3* ^+^ transcripts and a 60-fold increase of the SPAC1F8.02c transcript suggesting that these two flocculin genes and this uncharacterized glycoprotein gene may be responsible for the flocculent phenotype in this mutant. In contrast to the *yox1Δ* and *sre2Δ* mutants, *mbx2* ^+^ did not show differential expression in the *cbf11Δ* strain compared to wild type. However, the *cbf12* ^+^ transcript was upregulated 1.8-fold in the *cbf11Δ* strain. This suggests that *cbf11* ^+^ may regulate flocculation through *cbf12* ^+^, in agreement with previous reports of the antagonistic functions of *cbf11* ^+^ and *cbf12* ^+^ in this process [22].

We next determined whether the flocculation caused by the deletion of *yox1^+^*, *sre2^+^* or *cbf11^+^* was also dependent on *gsf2* ^+^. The absence of *gsf2* ^+^ was sufficient to abolish the flocculation in *yox1Δ*, *sre2Δ*, and *cbf11Δ* strains, even though *gsf2* ^+^ was not always the most highly-expressed flocculin gene (Figure 7A and 7C, Table S14). Taken together, these results suggest that the expression of the dominant flocculin Gsf2 is responsible for the bulk of flocculation observed in *yox1Δ*, *sre2Δ* and *cbf11Δ* strains.

### The role of flocculation by Adn2 and Adn3 is influenced by genes encoding cell wall–modifying enzymes and *gsf2^+^*

**Figure 8:**
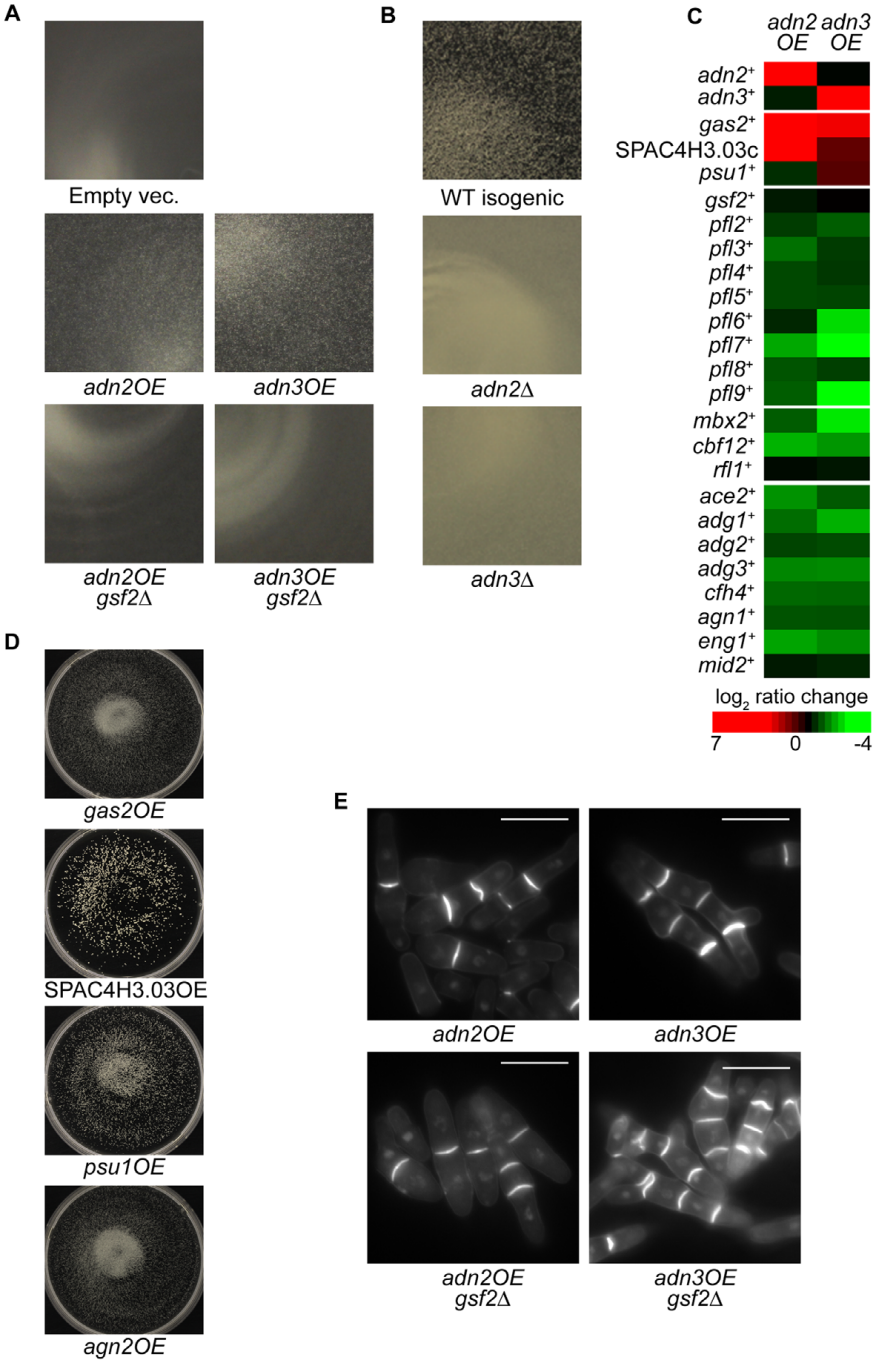
Regulation of flocculation by Adn2 and Adn3 is dependent on *gsf2^+^* and cell wall–remodeling genes. (A) Overexpression of *adn2^+^* or *adn3^+^* triggers weak flocculation, which is abrogated in the *gsf2*Δ background. The mutant strains were cultured for 3 days in EMM minus thiamine medium. Approximately 1/8 of the petri-dish was magnified and shown for each strain. (B) Deletion of *adn2* ^+^ or *adn3* ^+^ prevents flocculation in flocculation-inducing medium. (C) Several cell wall-remodeling genes were upregulated while the majority of *pfl^+^* genes and known target genes of Ace2 (bottom panel) were downregulated upon *adn2^+^* or *adn3^+^* overexpression. Microarray expression profiling was performed on *nmt1*-driven *adn2OE* and *adn3OE* strains and induced in EMM minus thiamine medium. The color bar reflects relative expression ratios between experimental and control strains. (D) Overexpression of the cell wall-remodeling genes *agn2^+^*, *psu1^+^*, *gas2^+^* and SPAC4H3.03c triggers flocculation. These overexpression strains were cultured for total of 5 days (subcultured on third day into fresh medium) in EMM minus thiamine medium. (E) The multiseptation phenotype resulting from *adn2^+^* or *adn3^+^* overexpression is not dependent on *gsf2^+^*. The strains were induced for 34 hours in EMM minus thiamine medium and stained with calcofluor white. Scale bar, 10 µm.

The transcription factor genes *adn2^+^* and *adn3^+^* are orthologous to *Sacc. cerevisiae FLO8* (http://www.pombase.org/) and exhibit defects in invasive growth and cell-to-surface adhesion when deleted during nitrogen starvation [29]. From our screens, we discovered that the overexpression of *adn2^+^* and *adn3^+^* triggered minor flocculation while loss of *adn2^+^* and *adn3^+^* prevented flocculation in flocculation-inducing medium (Figure 8A and 8B, respectively). The flocculent phenotype of *adn2OE* and *adn3OE* strains was disrupted by the addition of galactose (data not shown). To identify the target genes of Adn2 and Adn3 that are involved in flocculation, expression microarray profiling was performed on *nmt1*-driven *adn2OE* and *adn3OE* strains (Tables S11 and S12). Surprisingly, *gsf2* ^+^ transcript levels were relatively unchanged and the majority of *pfl^+^* genes were downregulated in both overexpression strains (Figure 8C). Consistent with these results were the observations that *mbx2* ^+^ and *cbf12* ^+^ transcripts were downregulated greater than 2-fold in both *adn2OE* and *adn3OE* strains, whereas *rfl1* ^+^ transcript levels were not differentially regulated (Figure 8C). Therefore, it appeared that the flocculent phenotype of *adn2^+^* and *adn3^+^* overexpression could not be attributed to the *pfl^+^* genes identified in this study. These results led us to consider that perhaps the expression of other genes besides these encoding for flocculins could be responsible for triggering flocculation in *adn2OE* and *adn3OE* strains.

Interestingly, some of the aforementioned cell wall-remodeling enzymes (*gas2* ^+^, *psu1* ^+^ and SPAC4H3.03c) were also highly upregulated in both *adn2OE* and *adn3OE* strains (Figure 8C, Table S13). For example, *gas2^+^* and SPAC4H3.03c were the highest induced genes in the *adn2OE* strain (17.9 and 36.8-fold, respectively) and also appeared within the top 20 most induced genes in the *adn3OE* strain. These genes were also induced in the *mbx2OE* strain except for *psu1* ^+^ (Figure 2A). Overexpression analysis was subsequently carried out to determine if these genes possessed some role in flocculation. Although *agn2^+^* was not upregulated in the *adn2OE* and *adn3OE* strains, it was included in the overexpression analysis because it was the second most induced gene (91-fold), as well as detected by ChIP-chip in the *mbx2OE* strain. Indeed, the single overexpression of these four genes resulted in flocculation after 5-days (including 3^rd^ day sub-culturing into fresh medium) in medium lacking thiamine, implicating the involvement of these cell wall-remodeling enzymes in flocculation (Figure 8D, Table S14). Since deletion of *adn2^+^* and *adn3^+^* results in defects of invasive growth and cell-to-surface adhesion in response to nitrogen starvation, we wanted to determine if the single overexpression of *gas2^+^*, *agn2^+^*, *psu1^+^* and SPAC4H3.03c could cause enhancement of these processes. We discovered that the single overexpression of these four cell wall-remodeling genes increased cell-to-surface adhesion, but not invasive growth relative to wild type under the nitrogen-deprivation condition (Figure S1). Because *gsf2^+^* encodes the dominant flocculin, we also investigated whether the flocculation caused by *adn2^+^* and *adn3^+^* overexpression was dependent on *gsf2^+^*. Deletion of *gsf2^+^* completely abrogated the flocculation in *adn2OE* and *adn3OE* strains (Figure 8A, Table S14).

In addition, the *adn2OE* and *adn3OE* strains exhibited cell separation defects such as the formation of multisepta and forkhead phenotypes (Figure 8E). The cell separation defect was more severe when *adn3^+^* was overexpressed. We next determined whether the putative target genes involved in the flocculation of *adn2OE* and *adn3OE* strains also played a role in the multisepta phenotype. Overexpression of *adn2^+^* and *adn3^+^* in the *gsf2*Δ background did not alter the multisepta phenotype (Figure 8E), while the overexpression of *gas2* ^+^, SPAC4H3.03c, *psu1* ^+^ and *agn2^+^* did not lead to formation of multisepta (data not shown). These results suggest that Adn2 and Adn3 may regulate cell separation and flocculation independently through different sets of target genes. Our microarray expression data suggests that Adn2 and Adn3 may control cell separation through *ace2^+^*, which encodes a major transcriptional activator of this process (Alonso-Nuñez *et al.*, 2005). Overexpression of *adn2^+^* and *adn3^+^* resulted in the down-regulation of *ace2^+^* and many of its known target genes such as *adg1* ^+^, *adg2* ^+^, *adg3* ^+^, *cfh4* ^+^, *agn1* ^+^, *eng1* ^+^, and *mid2* ^+^ by 1.5 to 3.4-fold (Figure 8C).

In summary, the regulation of flocculation by *adn2* ^+^ and *adn3* ^+^ is likely mediated by the induction of genes encoding the cell wall-remodeling enzymes Gas2, SPAC3H3.03c, and Psu1. The regulation of these genes is independent from Mbx2 because *mbx2^+^* was downregulated in the *adn2OE* and *adn3OE* strains. Although *gsf2^+^* transcript level was not significantly upregulated by *adn2^+^* and *adn3^+^* overexpression, it was sufficient to abrogate the flocculation when deleted. However, it is possible that other cell surface glycoprotein genes not investigated in this study but were upregulated may also play a significant role in the flocculation function of *adn2* ^+^ and *adn3* ^+^.

## Discussion

In this study, we have deciphered a significant portion of the transcriptional-regulatory network governing flocculation in *S. pombe*. To date, few transcription factors and their target genes that function in flocculation have been identified. The MADS box transcription factor Mbx2 positively regulates flocculation by induction of the flocculin gene *gsf2^+^*, while the CSL transcription factors Cbf11 and Cbf12 repress and activate flocculation, respectively, but their target genes are not known [21], [22]. We have substantially expanded our limited knowledge of the flocculation transcriptional-regulatory network by the identification of several novel transcriptional activators (Adn2 and Adn3) and repressors (Rfl1, Yox1 and Sre1), and their putative target genes that function in flocculation. In addition, novel target genes of Mbx2, Cbf11 and Cbf12 were identified. The putative target genes of the transcription factors implicated in flocculation encode for several cell surface glycoproteins (*gsf2^+^* and *pfl2* ^+^–*pfl9* ^+^) and cell wall-remodeling enzymes (*agn2^+^*, *psu1^+^*, SPAC4H3.03c and *gas2^+^*). These target genes were sufficient to trigger flocculation when overexpressed. Moreover, instances of regulation between transcription factors (Rfl1 repression of *mbx2^+^*), as well as positive (*mbx2^+^* and *cbf12^+^*) autoregulation were detected within the flocculation network.

Mbx2 and Rfl1 appeared to be the major positive and negative regulators of flocculation, respectively, based on the largest flocs observed in the *mbx2OE* and *rfl1*Δ strains compared to the other flocculent mutants in this study. Our initial efforts to identify the target genes of Mbx2 and Rfl1 revealed several putative flocculin genes that were strikingly upregulated in the *mbx2OE* and *rfl1*Δ flocculent mutants. Previously, Gsf2 was the only *S. pombe* flocculin demonstrated to be directly involved in flocculation, and its transcription was influenced by the activity of Mbx2 [20], [21]. Similar to these studies, we also found that overexpression of *gsf2^+^* triggers flocculation while loss of *gsf2^+^* abrogates the flocculent phenotype of several mutants including *mbx2OE*. Here, we identified an additional eight flocculin genes (*pfl2* ^+^–*pfl9^+^*) as putative target genes of Mbx2. Seven of these target genes (*pfl3* ^+^–*pfl9^+^*) were reported to contain tandem repeats found in fungal adhesins, while *pfl2* ^+^ is a sequence orphan predicted to encode a GPI-anchored protein [23], [27]. Seven *pfl^+^* genes (*gsf2* ^+^/*pfl1^+^*, *pfl3^+^*, *pfl4^+^ and pfl6^+^*–*pfl9^+^*) have been reported to be upregulated in loss-of-function flocculent mutants of Cdk8 module genes (*cdk8* ^+^/*srb10* ^+^, *med12* ^+^/*srb8* ^+^) suggesting that the transcriptional repression of these putative flocculin genes may be controlled by Mediator [24]. The transcriptional repression of flocculin genes by Mediator may not be direct, but could be through *mbx2^+^* since its expression is highly upregulated in the *cdk8* kinase-mutant and *med12Δ* strain (9 and 13-fold increase, respectively, within top 11 up-regulated genes, found in supplementary data [24]). This proposed role of Mediator appears conserved in *Sacc. cerevisiae* as *FLO* genes are similarly upregulated in *cdk8* mutants [38]. Despite these observations, no direct evidence has been shown aside from the *gsf2* ^+^ study by Matsuzawa *et al.* [20] that the *pfl^+^* gene products are actually flocculins. We have shown that this is indeed the case as single and double overexpression of the *pfl^+^* genes is sufficient to trigger flocculation and that this flocculation is galactose-specific.

The degree of flocculation triggered by single overexpression of the *pfl^+^* genes varied, with *gsf2^+^* and *pfl9^+^* producing the largest and smallest flocs, respectively (the *pfl* numbers correspond roughly to the degree of flocculation upon overexpression). This result indicates that *gsf2^+^* encodes the most dominant flocculin compared to the other *pfl^+^* genes. In agreement are the observations that only deletion of *gsf2^+^* and not the other *pfl^+^* genes prevented flocculation in flocculation-inducing medium, and reduced the constitutive flocculent phenotype to the greatest extent of all the transcription factor mutants examined in this study. Moreover, the strength of the flocculins was directly correlated with the amount of reduction in *mbx2OE* flocculation observed in the various *pfl* deletion backgrounds (Figure 2B). These observations are similar in *Sacc. cerevisiae*, where overexpression of *FLO1* produces the strongest flocculation compared to *FLO5*, *FLO9*, *FLO10* and *FLO11* [8],[12]. Furthermore, the flocculation mediated by *pfl^+^* genes was additive as observed in our double deletion and co-overexpression experiments (Figure 2B and Figure 3B). These results suggest that the varying strengths of flocculation exhibited by *S. pombe* strains could be attributed to the upregulation of different combinations of *pfl^+^* genes.

**Figure 9:**
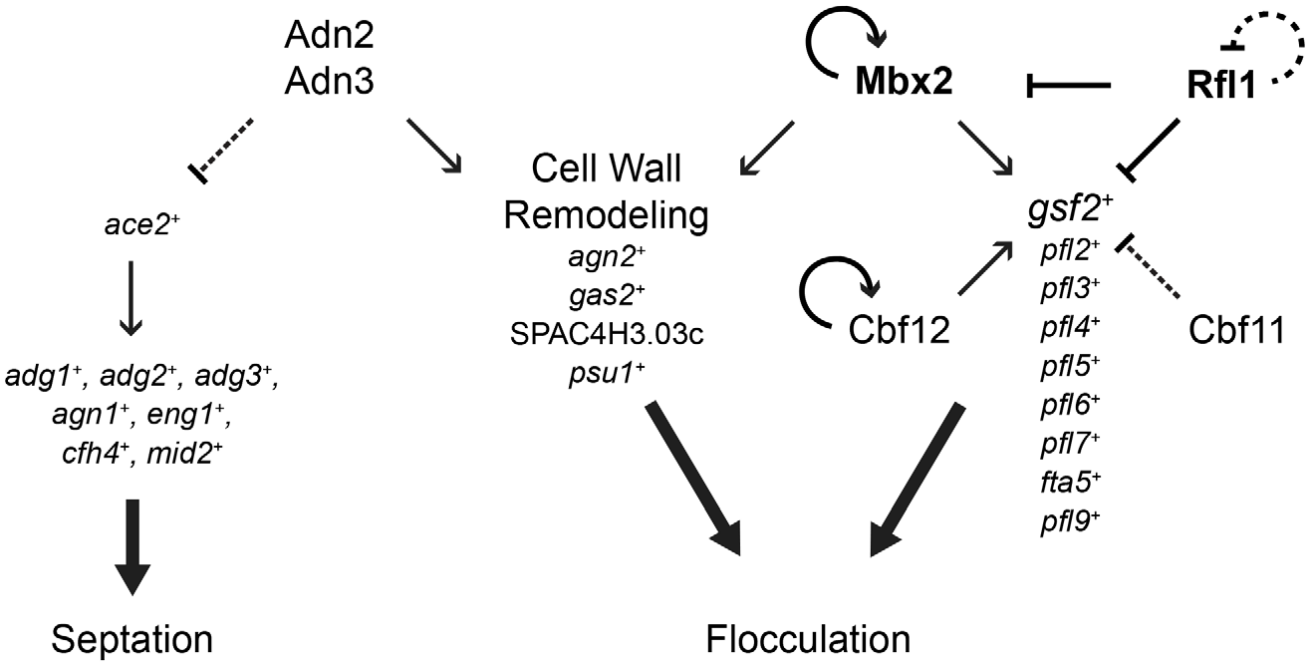
A model of the transcriptional-regulatory network of flocculation in *S. pombe*. Interactions of transcriptional activation and repression are indicated by arrows and bars, respectively. The major regulators of flocculation, Mbx2 and Rfl1 are in bold. Dash lines denote transcription factor-target gene interactions that may not be direct. See main text for a detailed description of the transcriptional-regulatory network.

We identified Rfl1, an uncharacterized Zn(2)-Cys(6) transcription factor as a novel repressor of flocculation in fission yeast. The repression of flocculation by Rfl1 appears to be primarily mediated by the inhibition of *gsf2^+^* expression since loss of *gsf2^+^* can abrogate the constitutive flocculent phenotype of the *rfl1*Δ mutant. Rfl1 represses *gsf2^+^* either directly by association with its promoter or indirectly by inhibition of *mbx2* ^+^ transcription, thereby forming an inhibitory feed-forward loop (coherent type 2) within the transcriptional-regulatory network (Figure 9). These results indicate that Mbx2 and Rfl1 are opposing transcription factors, and the latter inhibits *mbx2* ^+^ and *gsf2^+^* expression under non-inducing conditions of flocculation.

Aside from its role in flocculation, Rfl1 may have a role in regulating genes involved in carbohydrate metabolism such as glycolysis and gluconeogenesis. Rfl1 appeared to be associated with promoters of genes enriched in glucose catabolic and metabolic processes (p-values = 0.00092 and 0.00269, respectively) including *adh1* ^+^, *hxk2* ^+^, *pfk1* ^+^, *tpi1* ^+^, *adh4* ^+^, *pgi1* ^+^, *gpd3* ^+^, *tdh1* ^+^, *pgk1* ^+^, *fba1* ^+^, *eno101* ^+^, *pyr1* ^+^, SPCC794.01c (predicted glucose-6-phosphate 1-dehydrogenase), SPBC2G5.05 (predicted transketolase) and SPBC660.16 (phosphogluconate dehydrogenase). Most of these genes with the exception of *fba1* ^+^, *eno101* ^+^, SPCC794.01, and SPBC660.16 were upregulated 1.2 to 26-fold in the *rfl1*Δ strain (Table S4). From these data, we speculate that Rfl1 could serve as a negative transcriptional regulator of several enzymes involved in the glycolysis and gluconeogenesis. Because flocculation and invasive growth are associated with nutritional limitation, Rfl1 may coordinate the expression of genes involved in flocculation and carbohydrate metabolism in fission yeast.

Previously, the CSL proteins Cbf11 and Cbf12 were shown to exhibit antagonistic roles in flocculation [39]. Overexpression of *cbf12^+^* or loss of *cbf11^+^* triggers flocculation. However, none of their target genes have been identified. We present supportive evidence that Cbf12 induces flocculation by directly activating the transcription of *gsf2^+^*. In addition, *gsf2^+^* expression is up-regulated approximately 2.4-fold in the *cbf11Δ* strain suggesting that the repressive flocculation function of Cbf11 may also be directly mediated through *gsf2^+^*. The activation and repression of *gsf2^+^* transcription by Cbf12 and Cbf11, respectively, may occur by competitive binding to promoter sites since both transcription factors have been shown to interact with a canonical CSL consensus sequence *in vitro* [39]. Several putative sites with six out of seven nucleotide match to the canonical CSL consensus sequence are located within 900 base pairs of the *gsf2^+^* promoter (data not shown). Further experimentation would be required to verify this proposed mechanism of *gsf2^+^* transcriptional regulation by Cbf11 and Cbf12. It is likely that *cbf12* ^+^ plays a lesser role in activating flocculation compared to *mbx2* ^+^ since the floc size resulting from *cbf12* ^+^ overexpression is considerably smaller than the *mbx2OE* strain. Also unlike *mbx2^+^*, deletion of *cbf12* ^+^ is not sufficient to abrogate the flocculation of the *rfl1*Δ strain (data not shown). These data suggest that the flocculent phenotype of the *cbf12Δ rfl1*Δ double mutant is probably caused by the presence of *mbx2* ^+^ activity.

CSL transcription factors are components of the conserved Notch signaling pathways in metazoans which primarily function in cell-to-cell communication during development [40]. Although multiple fungal CSL proteins have been discovered, their exact roles remain unclear in unicellular organisms [39]. Flocculation has been described as a manifestation of social behaviour in yeast with a purpose of enhancing survival under stressful conditions [3]. Therefore, it is conceivable that CSL transcription factors originated as regulators of this primitive form of cell-to-cell communication, and later evolved into the metazoan Notch signaling pathway.

We also discovered novel functions of the Yox1 and Sre2 transcription factors in the repression of flocculation. Loss of *yox1^+^* or *sre2* ^+^ results in a mild flocculent phenotype. The Yox1 homeodomain transcription factor functions as a repressor of MBF (Mlu1 binding factor) target genes to prevent their inappropriate expression at the end of S-phase [36]. Transcriptional repression of MBF target genes is mediated by the direct interaction of Yox1 and Nrm1 to the MBF complex [41]. Deletion of *yox1^+^* causes a cell cycle delay and results in elevated constitutive expression of MBF gene targets [36]. Similarly, these genes (e.g. *cdc18* ^+^, *cdc22* ^+^, *cdc10* ^+^, *cdt1* ^+^, *cdt2* ^+^, *cig2* ^+^ and *nrm1* ^+^) were also found to be upregulated 2.2 to 6.4-fold in our *yox1*Δ microarray expression data (Table S8). We found that the flocculent phenotype of the *yox1*Δ strain is also dependent upon *gsf2^+^*. However, the *pfl* ^+^ genes including *gsf2^+^* were not highly expressed in the *yox1*Δ strain. One possible explanation why *pfl1* ^+^ genes were not highly expressed in the *yox1*Δ strain is that our experiments were performed under asynchronous culturing conditions, and therefore, the upregulation of *pfl^+^* genes including *gsf2^+^* could have been obscured if their expressions were periodically controlled in the vegetative cell cycle. However, it is unlikely that Yox1 regulates the *pfl* ^+^ genes directly because previous chIP-chip analysis did not detect binding of Yox1 to the promoters of *pfl* ^+^ genes [36]. Although there has been solid evidence linking yeast morphogenesis events such as pseudohyphal and hyphal growth to cell cycle regulators [42]–[47], the relationship between cell cycle control and flocculation remains unclear. A flocculation function for *yox1^+^* has not been reported in other yeasts. However, disruption of *YOX1* in the *Sacc. cerevisiae* ∑1278b strain inhibited filamentous invasive growth, a process usually associated with flocculation during nutritional limitation, while deletion of *C. albicans NRM1* reduced flocculation [48], [49].

Sre2 is an uncharacterized membrane-tethered helix-loop-helix transcription factor predicted to be an ortholog of mammalian SREBP-1a, which is responsible for the transcriptional activation of genes needed for uptake and synthesis of cholesterol, fatty acids, triglycerides, and phospholipids [50]. While *sre1* ^+^, a paralog of *sre2* ^+^ has been shown to function in the transcriptional activation of sterol-biosynthetic and hypoxic-adaptation genes, there has been no direct evidence that *sre2* ^+^ plays similar biological roles [37]. Loss of *sre2* ^+^ results in the upregulation of *gsf2* ^+^, *pfl3* ^+^ and *fta5* ^+^ transcripts (3.76, 2.51 and 3.08-fold, respectively) (Figure 7C, Table S9) which may contribute to its flocculent phenotype. The *sre2*Δ flocculent phenotype requires *gsf2* ^+^ activity and is independent of *mbx2* ^+^ and *cbf12* ^+^ since these transcripts are downregulated in the deletion mutant.

In addition, the microarray expression profiles of *yox1*Δ and *sre2*Δ strains displayed similar differential gene expression despite the supposedly different functions of these transcription factors (Figure 7B). Mitochondrial genes were found to be highly upregulated in both deletion mutants (Tables S8 and S9). This occurrence may not be unexpected for Sre2 if it has a similar role in hypoxia as Sre1 where mitochondrial function is probably impaired [37]. It is currently not clear whether the mitochondrial genes are direct targets of Yox1 and Sre2 or induced in response to an altered physiological state in the deletion mutants. Interestingly, mitochondrial activity has been reported to be important for flocculation and invasive growth in *Sacc. cerevisiae* [48], [51]. Disruption of mitochondrial activity has been shown to alter the synthesis and structure of the cell wall, possibly by interfering with the interactions of flocculins and their substrates [52]. Based on these observations, the flocculent phenotype of *yox1*Δ and *sre2*Δ strains could be partially the result of enhanced mitochondrial activity from the upregulation of mitochondrial genes.

A genome-wide systematic deletion screen previously uncovered a cell-to-surface adhesion function that is sensitive to the presence of galactose for the Adn2 and Adn3 transcription factors [29]. Here, we discovered that *adn2^+^* and *adn3^+^* have additional functions in flocculation. Overexpression of *adn2^+^* and *adn3^+^* induced minor flocculation while loss of these genes prevented flocculation in inducing glycerol medium (Figure 8A and 8B). However, the flocculent phenotype of the *adn2OE* and *adn3OE* strains appeared to be primarily caused by the differential regulation of genes encoding cell wall-remodeling enzymes rather than flocculins. Several genes encoding cell wall-remodeling enzymes (*gas2* ^+^, *agn2* ^+^, *psu1* ^+^ and SPAC4H3.03c) were highly induced when *mbx2^+^*, *adn2^+^* or *adn3^+^* was overexpressed. In the *adn2OE* strain, *gas2* ^+^ and SPAC4H3.03c were the most highly induced genes (17.9 fold and 36.8-fold, respectively) (Figure 8C, Table S11) while in the *adn3OE* strain, these two genes and *psu1* ^+^ appeared within the top 20 up-regulated genes (Table S12). Similarly, in the *mbx2OE* strain, *gas2* ^+^, *agn2* ^+^, and SPAC4H3.03c appeared within the top 100 up-regulated genes (greater than 3.7-fold increase, Figure 2A). We found that the single overexpression of these four genes could trigger flocculation (Figure 8D). Cell wall remodeling is an essential process for proper growth and adaptation to environmental stresses in yeast cells. Part of the cell wall-remodeling process involves the dissolution of sugar moieties in the glucan layer and elongation of glucan chains by glycoside hydrolases and glycosyltransferases, respectively. Among these four genes, three (*agn2* ^+^, *psu1* ^+^ and SPAC4H3.03c) encode for glycoside hydrolases while the fourth (*gas2* ^+^) encodes for a glycosyltransferase. Agn2 is an endo-(1,3)-α-glucanase that hydrolyzes (1,3)-α-glucans of the ascus wall for ascospore release [53], [54]. Although *agn2* ^+^ function appears only specific for sporulation, its ectopic expression could alter the cell wall structure during vegetative growth by inappropriate hydrolysis of (1,3)-α-glucan. Similarly, inappropriate glucan hydrolysis of the cell wall could be occurring as a result of ectopic expression of SPAC4H3.03c which encodes a putative (1,4)-α-glucanase (Hertz-Fowler *et al.*, 2004). Psu1, which exhibits close homology to the members of the SUN family in *Sacc. cerevisiae* and *C. albicans*, as well as the BglA beta glucosidase of *C. wickerhamii*, has an essential function in cell wall synthesis [55]. Loss of *psu1* ^+^ activity conferred resistance to (1,3)-β-glucanase suggesting that Psu1 may influence the amount or structure of (1,3)-β-glucan in the cell wall [55]. In addition, the (1,3)-β-glucanosyltransferase Gas2 has been shown to lengthen glucan chains during cell wall assembly and its overproduction is able to suppress the cell wall defect and lethality of *gas1*Δ cells [56]. Then how does the overexpression of these cell wall-remodeling genes trigger flocculation in *S. pombe* cells? The expression of flocculin genes during vegetative growth is not well characterized in yeasts, but studies in *Sacc. cerevisiae* indicate that flocculin synthesis and insertion into the cell wall initiate in early exponential phase prior to the onset of flocculation during stationary phase [57]. This suggests that the flocculins are already present in the cell wall, but cannot induce flocculation because of inaccessibility to cell surface oligosaccharides. We speculate that the restructuring of the β-glucan layer during cell wall remodeling may result in the rearrangement of flocculins that enhances galactose oligosaccharide binding, thereby promoting flocculation. Several lines of evidence in *Sacc. cerevisiae* support this hypothesis. First, alteration of cell wall structure by disruption of *PKC1* activity results in flocculation [58]. Second, heat shock induces flocculation and regulation of cell wall-remodeling genes via the Hsf1 transcription factor [57], [59]. Currently, we cannot rule out that *agn2* ^+^, *psu1* ^+^, SPAC4H3.03c and *gas2* ^+^ are the only cell wall remodeling enzymes that can trigger flocculation when overexpressed. Other genes with potential functions in cell wall modification and integrity such as *gas4* ^+^, *gma12* ^+^, *meu7* ^+^, *agl1* ^+^, *meu10* ^+^ and *mde5* ^+^ were also detected as putative target genes of Mbx2 (Table S2). In contrast, there was little change in *gsf2^+^* transcript levels in the *adn2OE* or *adn3OE* strains compared to the empty vector control. However, the flocculation triggered by *adn2^+^* and *adn3^+^* overexpression was abrogated in a *gsf2*Δ background indicating that *gsf2^+^* was indispensible for this process (Figure 8A). Altogether, these results suggest that Gsf2 is likely expressed in the cell wall as an inactive flocculin, and the cell wall remodeling resulting from *adn2^+^* and *adn3^+^* overexpression alters the arrangement of Gsf2 and possibly other flocculins that now becomes favorable for flocculation.

The single overexpression of the cell wall-remodeling genes triggered flocculation to a greater extent than the *adn2OE* and *adn3OE* strains. A possible explanation for the different degrees of flocculation between the transcription factor and its target genes could be that overexpression of *adn2^+^* and *adn3^+^* causes reduced fitness due to toxicity effects associated with a greater misregulation of genes compared to the aberrant production of a single enzyme. Consistent with this theory is that *adn2OE* and *adn3OE* strains exhibited additional phenotypes including septation defects (Figure 8E) which were not observed when *gas2* ^+^, *agn2* ^+^, *psu1* ^+^ and SPAC4H3.03c were overexpressed (data not shown). Furthermore, a systematic overexpression analysis of 5280 genes in *Sacc. cerevisiae* revealed that genes encoding for transcription factors, signalling molecules and cell cycle regulators were more likely to cause reduced fitness [60].

In *S. pombe*, cell separation involves the transcriptional activation of *adg1* ^+^, *adg2* ^+^, *adg3* ^+^, *agn1* ^+^, *eng1* ^+^, *cfh4* ^+^ and *mid2^+^* by the Ace2 transcription factor, which is in turn regulated by the Sep1 forkhead transcription factor [61]–[64]. We discovered that the *adn2OE* or *adn3OE* strains displayed multisepta and forkhead phenotypes similar to loss-of-function mutations of these cell separation genes. The cell separation defect in *adn2OE* and *adn3OE* strains is likely due to the downregulation of *ace2* ^+^ transcription since *ace2^+^* and its target genes were substantially downregulated in these strains (Figure 8C). However, *sep1^+^* transcript levels remained unchanged in the *adn2OE* and *adn3OE* strains indicating that their involvement in cell separation phenotype could be either downstream of *sep1* ^+^ or parallel to the *sep1* ^+^ pathway. In additional to its flocculation role, Adn2 and Adn3 appear to have a separate function in cell separation perhaps by directly or indirectly repressing *ace2^+^* transcription. Experiments are planned in the future to address these possibilities.

Interestingly, we also found some evidence that supports a role of Mbx2 and Cbf12 in cell separation perhaps through repression of *ace2^+^* activity. Overexpression of *mbx2^+^* and *cbf12^+^* results in significant down-regulation of all seven Ace2 target genes approximately 1.5 to 3.4-fold relative to the empty vector control. (Tables S2 and S6). The *mbx2OE* strain indeed showed septation defects but were slightly different in nature than the *adn2OE* and *adn3OE* strains with less multi-septation and more mislocalization of septum material (data not shown). Moreover, overexpression of *cbf12^+^* has been reported to produce multisepta phenotypes albeit at a low frequency [39]. These observations indicate the possible existence of crosstalk between flocculation and cell separation pathways mediated by the Mbx2, Cbf12, Adn2 and Adn3 transcription factors (Figure 9).

A comparison between the flocculation network of budding and fission yeast revealed both conserved and divergent features within the transcriptional circuitry. In *Sacc. cerevisiae*, the positive and negative transcriptional controls of the dominant flocculin gene *FLO1* by Flo8p or Mss11p, and Sfl1p, respectively, draw parallel to *gsf2^+^* regulation by the Mbx2-Rfl1 and Cbf12-Cbf11 opposing transcription factors in *S. pombe* [16], [19]. The conservation of the flocculin genes between these two yeasts is apparent among these transcription factors. Similar to Mbx2, Mss11p and Flo8p appear to activate multiple flocculin genes (*FLO1*, *FLO9* and *FLO11*), while the latter may also regulate genes encoding cell wall enzymes (*STA1* and *SGA1* which both encode glycoside hydrolases) [9], [16]. The putative target genes of the Sfl1 repressor have been reported to include *FLO1* and *FLO11* [19], [65]. In contrast to the conservation of the flocculin genes, the types of transcription factors involved in flocculation are quite different between the two yeasts. Mbx2 belongs to the MADS box family, while the DNA-binding domains of Flo8p and Mss11p have not been defined. In addition, the Sfl1p and Rfl1 repressors contain the heat shock factor and Zn(2)-Cys(6) DNA-binding domains, respectively. Moreover, CSL transcription factors (Cbf11/Cbf12) are not found in *Sacc. cerevisiae*. These observations would imply that the cis-regulatory elements controlling transcription of the flocculin genes have likely undergone considerable rewiring within the transcriptional-regulatory network between the two yeasts. However, it was recently demonstrated that heterologous expression of Flo8p and Mbx2 could induce *gsf2^+^* and *FLO1* transcription in fission and budding yeast, respectively [20], [21]. Therefore, despite the divergent types of transcription factors controlling flocculin gene expression in the two yeasts, there may be some degree of conservation among the cis-regulatory sequences.

Although the transcription factors regulating flocculation appear to be quite different between the two yeasts, the downstream transcriptional events involved in the repression of flocculin genes are likely to be conserved. Disruption of genes encoding the Ssn6p-Tup1p general corepressor or the Cdk8 module of Srb/Mediator complex have been shown to cause upregulation of flocculin genes and constitutive flocculation in *S. pombe* and *Sacc. cerevisiae* [65]. In the latter yeast, Sfl1p represses *FLO1* and *FLO11* transcription through physical interactions with Ssn6p and Srb proteins (Srb8p, Srb9p and Srb11p) [19], [65], [66]. Moreover, Sfl1p has been reported to repress *FLO8* in *Saccharomyces diastaticus*. The observation that Sfl1p can repress *FLO1* transcription directly and indirectly through *FLO8* seems very similar to the inhibitory feed-forward regulation of *gsf2^+^* by Rfl1 in *S. pombe*. If these connections are truly analogous, then there is a possibility that Rfl1 repression could also be mediated through physical interactions with the Cdk8 module proteins. In the *srb10^−^* mutant, *gsf2^+^* and *mbx2^+^* expression are upregulated suggesting that its flocculent phenotype could be caused by a failure to repress *mbx2^+^* transcription [24]. In addition, the flocculent phenotype of *tup11* ^+^/tup*12* ^+^ mutants [25] and the abrogation of *lkh1*Δ flocculation in the absence of *mbx2* ^+^ [21] supports the role of Tup11/12 corepressor in Mbx2-Rfl1-mediated flocculation. Taken together, we speculate that Srb10 and Tup11/12 activity and binding may be required for Rfl1-mediated repression of *gsf2^+^* and *mbx2^+^*. Future experiments focusing on the interactions between Rfl1, Tup11/12 and Srb8-10 in relation to flocculation would provide clarification to our speculation.

Our analyses of the transcription factors implicated in flocculation of *S. pombe* revealed the possible existence of several network motifs including positive autoregulation of *mbx2^+^* and *cbf12^+^* and regulation of *gsf2^+^* by a inhibitory feed-forward loop (coherent type 2). The latter involves the Rfl1 transcriptional repression of *gsf2^+^* directly and indirectly by inhibition of *mbx2^+^* expression. Autoregulatory motifs have not been detected so far for *FLO8*, *MSS11* and *SFL1*. The discovery of these network motifs in *S. pombe* suggests that the transcriptional inhibition of *gsf2^+^* could occur more rapidly than its transcriptional activation. Experimental and modeling studies have proposed that positive and negative autoregulation of transcription factors generate slow and fast response times, respectively, within a transcriptional-regulatory network [67]. Under positive autoregulation, the synthesis rate of the transcription factor is initially slow at low concentrations, but increases as the concentration of the transcription factor reaches the activation threshold of the promoter, while negative autoregulation accelerates the attainment of steady state levels of the transcription factor [67]. Moreover, the inhibitory feed-forward motif of Rfl1 seems to indicate that repression of *gsf2^+^* expression likely happens in a shorter period compared to its activation. Altogether, these data suggest that the onset of flocculation may occur gradually while repression of the flocculation pathway is a much faster process. Consistent with this speculation is the observation that it requires several days for wild-type *S. pombe* cells to undergo flocculation when grown in inducing medium.

In summary, we have provided an initial and substantial view of the transcriptional-regulatory network governing flocculation in *S. pombe*. Found within this network are the master regulators Mbx2, Cbf12, Adn2 and Adn3, which are able to trigger flocculation when overexpressed by the activation of their target genes encoding for flocculins and cell wall-remodeling enzymes. In addition, several repressors including Rfl1 were uncovered that play a major role in the regulation of these target genes. However, significant gaps of knowledge surrounding the transcriptional-regulatory network still remain. The environmental cues that impinge upon the activity of the positive and negative regulators, as well as the dynamics of transcription factor binding and regulation of target genes during the onset of flocculation remain to be elucidated. Also, although *gsf2^+^* encodes the dominant flocculin, it is currently unclear whether the other flocculins have nonessential or more specialized roles during flocculation. Detailed analyses of the temporal and spatial expression of the *pfl^+^* genes would be required to address these questions. Moreover, the exact mechanism of how other biological processes such as cell wall restructuring and mitochondrial function influence flocculation is unknown. Further studies to expand our knowledge of this transcriptional-regulatory network would provide a more comprehensive understanding of flocculation control and contribute to a valuable resource for the improvement of industrial yeast applications.

## Materials and Methods

### Yeast strains, media, and general methods

All strains used in this study are listed in Table S1 and were maintained on YES or EMM medium. Geneticin, nourseothricin, and thiamine hydrochloride were added to media at a concentration of 150 mg/L, 100 mg/L, and 15 µM, respectively. EMM medium was supplemented with amino acids when necessary at 225 mg/L each for adenine, leucine, and uracil. Matings were performed on SPAS medium. Wild type and deletion strains were assayed for flocculation in YEGlyEtOH (flocculation-inducing) medium containing 1% (w/v) yeast extract, 3% (v/v) glycerol, and 4% (v/v) ethanol. Overexpression strains containing ORFs under control of the *nmt1* or *nmt41* promoter were grown in EMM minus thiamine medium. Standard genetics and molecular biology techniques were performed as described in [68].

### Construction of deletion and GFP-tagged strains

A PCR-based stitching method was utilized to construct the deletion and epitope-tagged strains. For construction of deletion strains, ∼500 bp fragments upstream and downstream of the ORF and the KanMX6 or NatMX6 cassette were PCR-amplified and gel-purified. The 3′ end of the upstream fragment and 5′ end of the downstream fragment contained ∼25 bp homology to the selectable marker cassette sequence. Approximately equimolar amounts (∼40 ng) of each PCR fragment were combined and stitched together in a 20 µl PCR reaction (0.2 mM dNTPs and 0.4 units of Phusion HF DNA polymerase (New England Biolabs), and subjected to one cycle of 98°C (30 sec), 5 cycles of 98°C (15 sec), 60°C (1 min), and 72°C (1–2 min) and a final extension at 72°C (5 min). The stitched product was then amplified in a 50 µl PCR reaction by combining the entire stitched reaction with 6 nmol dNTPs, 0.6 units of Phusion HF DNA polymerase and 20 pmol each of the outer pair of primers and then subjected to one cycle of 98°C (30 sec), 30 cycles of 98°C (10 sec), 60°C (30 sec) and 72°C (2 min), and a final extension at 72°C (5 min). The amplified product was gel-purified and transformed into the appropriate strain by lithium acetate transformation. A similar strategy was used to construct GFP-tagged transcription factors under the control of the native promoter. To tag the transcription factor with GFP at the C-terminus, ∼500 bp upstream and downstream fragments flanking the stop codon and the GFP-KanMX6 cassette (amplified from pYM27 plasmid, [69]) were PCR-amplified for the stitching reaction as described above. To conserve the native promoter in the N-terminal GFP fusion of Mbx2, 1 kb upstream of the *mbx2* ^+^ start codon was amplified along with four other fragments for PCR stitching: (1) ∼500 bp upstream of the aforementioned 1 kb fragment; (2) ∼500 bp downstream of the *mbx2* ^+^ start codon; (3) KanMX6 cassette and; (4) the GFP ORF with its stop codon removed and a GDGAGL linker added (adapted from [70]). All five fragments contained ∼25 bp overlapping homology to their respective flanking fragments and were PCR-stitched as described above. Proper gene deletion and GFP tagging were confirmed by colony PCR screen and the resulting amplicons sequenced.

### Construction of overexpression strains

Genes were overexpressed with the *nmt1* promoter by cloning the entire ORFs of interest into the *pREP1* or *pREP2* vector. For ChIP-chip experiments, C-terminal triple HA-tagged Mbx2, Rfl1, and Cbf12 were expressed with the *nmt41* promoter by cloning the corresponding ORFs into *pSLF272* [71]. All the clones were PCR-confirmed, sequenced, and transformed into appropriate strains by the lithium acetate method. Expression of the HA-tagged proteins was verified by western blotting with anti-HA F-7 antibody (Santa Cruz Biotechnology, Santa Cruz, CA).

### Microarray expression profiling

Strains overexpressing the triple HA-tagged Mbx2, Rfl1, and Cbf12 were grown in 200 ml of EMM medium containing appropriate supplements without thiamine for 18-20 hr to induce the *nmt41* promoter. The empty vector control strain was cultured concurrently to a matching cell density of ∼8×10^6^ cells/ml prior to harvesting. The experimental culture was divided into two, each for ChIP-chip and microarray expression profiling while the control culture was only utilized in the latter. The expression profiling cultures were harvested by centrifugation (1800× g, 3 min, 20°C), followed by immediate freezing of the cell pellets in liquid nitrogen. Culturing of *adn2OE* and *adn3OE* strains were performed similarly except that these genes were driven by the *nmt1* promoter and were not epitope-tagged. For transcription factor deletion strains (*rfl1*Δ, *cbf11*Δ, *sre2*Δ, and *yox1*Δ), the mutant and an isogenic wild-type strain were concurrently grown in YES medium and harvested at a similar cell density as described above. Total RNA extraction, mRNA isolation, reverse transcription with aminoallyl-dUTP (Sigma-Aldrich, Oakville, ON), and Cy™3/Cy™5 (GE Healthcare, Buckinghamshire, UK) dye coupling of cDNA were performed with dye reversal as previously described [72]. Purified Cy™3- and Cy™5-labelled cDNA (1 µg in total) was hybridized onto custom-designed 8×15 K Agilent expression microarrays containing 60mer probes to all *S. pombe* ORFs in 2–3 times coverage per gene. The hybridization procedure was carried out according to the manufacturer's instructions (Agilent Technology, Santa Clara, CA) with the exception for the use of Human Cot-1 DNA. The microarrays were washed in 6× SSPE/0.005% sodium N-lauroylsarcosine at room temperature for 5 min followed by a second wash in pre-heated 42°C 0.6× SSPE for 2 min.

The microarrays were scanned with a GenePix4200A scanner (Molecular Devices, Sunnyvale, CA). The raw microarray data was lowess normalized [73] and the average log_2_ ratios with the corresponding t-test p values [74] from the dye-swap experiments were obtained using the R Bioconductor Limma package. Heat map images of the microarray expression and ChIP-chip data were constructed with Cluster 3.0 [75] and Java Treeview 1.1.6r2 [76]. The microarray expression data has been submitted to the NCBI Gene Expression Omnibus Database (GSE41730).

### ChIP–chip experiments and data analysis

Culturing of the HA-tagged transcription factor strains are described above. The culture was fixed by the addition of a final concentration of 1% formaldehyde and agitation for 30 min at room temperature. The formaldehyde was quenched by the addition of 2.5 M glycine to a final concentration of 125 mM and agitation for 5 min at room temperature. The cells were then centrifuged (800× g, 5 min, 4°C), washed twice in 25 ml 1× ice-cold PBS (137 mM NaCl, 2.7 mM KCl, 10 mM Na_2_HPO_4_, 1.8 mM KH_2_PO_4_ pH 7.4) and washed once with 2 ml ice-cold lysis buffer (50 mM NaCl, 50 mM HEPES-KOH pH 7.5, 0.1% SDS, 1% Triton X-100, 1 mM EDTA, 0.1% sodium deoxycholate and 1 tablet/50 ml Protease Inhibitor Cocktail (Roche Applied Science, Indianapolis, IN)). The cell pellet was resuspended in 1.6 ml lysis buffer and stored at −80°C.

The cell suspension was transferred to two 2 ml bead beating vials containing 800 µl of 0.5 mm Zirconia/Silica beads (BioSpec Products, Bartlesville, OK) and subjected to 3 cycles of alternating 2 min beating and 2 min incubation on ice with a Mini Beadbeater 16 (BioSpec Products, Bartlesville, OK). The lysed cells were collected by puncturing the bottom of the bead-beating vial with a flame-heated inoculating needle and placing the vial on a sonication tube nested in 10 ml disposable culture tubes prior to centrifugation (800× g, 3 min, 4°C). The cell pellet was resuspended, transferred to chilled microcentrifuge tubes, centrifuged (16,000× g, 15 min, 4°C) to remove unbound soluble proteins, and the resulting pellet resuspended in 800 µl of fresh lysis buffer in a sonication tube. Total cell lysate volume was adjusted to 2.2 ml with lysis buffer and subjected to 4 cycles of sonication and 1 min on ice incubation at 30% amplitude, 30 sec setting using a Sonic Dismembrator with a 1/8 tapered microtip probe (Thermo Scientific, Waltham, MA). The sonicated cell lysate was centrifuged (4600× g, 2 min, 4°C) and the supernatant stored at −80°C. The supernatant was tested to ensure that greater than 90% of the sonicated DNA was in the size range of 100 bp–1 kb by subjecting a sample (∼50 µl) of the supernatant to overnight reverse-crosslinking at 65°C and phenol-chloroform extraction, followed by gel electrophoresis of 3–5 µg of DNA. To immunoprecipitate the chromatin-bound transcription factor, 100–200 µl of Dynabeads conjugated with sheep anti-mouse IgG (Invitrogen Life Technologies, Carlsbad, CA) were washed twice in 800 µl ice cold 1× PBS-BSA (5 mg/ml BSA, 1× PBS), resuspended in 800 µl cold 1× PBS-BSA with 5 µg of anti-HA F-7 antibody (Santa Cruz Biotechnology, Santa Cruz, CA) and shaken gently for 2 hr at 4°C on a Labquake Tube Shaker (Thermo Scientific, Waltham, MA). The beads were washed twice in 1 ml cold deoxycholate buffer (100 mM Tris-HCl pH 8, 1 mM EDTA, 0.5% (w/v) sodium deoxycholate, 0.5% (v/v) NP-40, 250 mM LiCl) and twice in 1 ml cold lysis buffer. The beads were resuspended in 200 µl 1× PBS-BSA, combined with 400 µl of sonicated cell lysate, and shaken gently for 2 hr at 4°C. Four washes of 5 minutes each were next carried out: (1) 1.4 ml cold lysis buffer at 4°C; (2) 1.4 ml cold lysis buffer with 400 mM NaCl at 4°C; (3) 1.4 ml deoxycholate buffer at room temperature and; (4) 1.4 ml TE (pH 8) at room temperature. The transcription factor and bound DNA were eluted twice from the Dynabeads by incubating with 250 µl TES each (TE pH 8, 1% (w/v) SDS) at 65°C for 6 min. Dynabead washing and the supernatant collection were performed using DynaMag™^−2^ (Invitrogen, Carlsbad, CA). For the input DNA, 200 µl of the cell lysate was added to 300 µl TES. Both the immunoprecipitated and input cell lysates were incubated at 65°C overnight to reverse the DNA-protein cross-linking. Western blotting with anti-HA antibody was performed to confirm proper pull-down of the transcription factor.

For protein removal, both immunoprecipitated and input samples were incubated with 200 µg Proteinase K (Promega, Madison, WI) and 20 µg glycogen (Roche Applied Science, Indianapolis, IN) at 56°C for 2 hr. The DNA was then extracted by phenol-chloroform extraction, ethanol-precipitated overnight, washed once with 70% EtOH, resuspended in 42 µl TE containing 0.1 µg DNAse-free RNaseA (Roche Applied Science, Indianapolis, IN), and incubated for 30 min at 37°C.

Blunt ends were generated in the entire immunoprecipitate and input DNA samples with 1 unit of T4 DNA Polymerase (Invitrogen Life Technologies, Carlsbad, CA), 1× NEB Buffer #2 (New England Biolabs, Ipswich, MA), 5 µg NEB BSA, and 10 nmol dNTPs in a 110 µl reaction by incubation at 12°C for 20 min, followed by phenol-chloroform extraction and ethanol precipitation with 10 µg glycogen and 1/10 volume 3 M NaOAc. The DNA pellets were washed in 70% EtOH and resuspended in 25 µl water. Approximately 1/5 of precipitated input DNA was used in the subsequent ligation reaction as input DNA concentration was >100 times greater than that of immunoprecipitated DNA. For ligation of linkers to blunt ends, the resuspended DNA was incubated with 1000 units of concentrated T4 DNA Ligase (New England Biolabs, Ipswich, MA), 1× T4 DNA Ligase Buffer (Invitrogen Life Technologies, Carlsbad, CA), and 200 pmol annealed linker (15 µM Oligo #1 5′-GCGGTGACCCGGGAGATCTGAATTC-3′ and 15 µM Oligo #2 5′-GAATTCAGATC-3′ in 250 mM Tris) at 16°C overnight. The annealed linker and the ligation mix were kept on ice at all times prior to overnight incubation. The DNA was ethanol-precipitated, washed, and resuspended in 25 µl water as described above.

The ligated DNA was PCR-amplified by adding 15 µl of labeling mix (2 µl aa-dUTP dNTP mix containing 5 mM each dATP, dCTP and dGTP, 3 mM dTTP, and 2 mM aminoallyl-dUTP (Sigma-Aldrich, St. Louis, MO)), 1.25 µl 40 µM Oligo #1 (5′-GCGGTGACCCGGGAGATCTGAATTC-3′), 4 µl 10× ThermoPol Buffer (New England Biolabs, Ipswich, MA) and 7.75 µl water) in a PCR cycler paused at 55°C. A 10 µl enzyme mix containing 5 units of GoTaq DNA polymerase (Promega, Madison, WI), 0.001 units of Pfu Turbo DNA polymerase (Stratagene, La Jolla, CA), and 1× ThermoPol Buffer (New England Biolabs, Ipswich, MA) was added and the PCR proceeded with one cycle of 55°C (4 min); 72°C (5 min); 95°C (2 min) and 30 cycles of 95°C (30 sec); 55°C (30 sec); 72°C (1 min), followed by a final extension at 72°C (4 min). The PCR products were purified (QIAGEN, Valencia, CA) with a few modifications: (1) buffer PE was replaced with phosphate wash buffer (5 mM KPO_4_ pH 8.5, 80% ethanol) and (2) buffer EB was replaced with phosphate elution buffer (4 mM KPO_4_ pH 8.5). A sample of the purified PCR product was run on an agarose gel to check for fragment sizes ranging between 100 bp and 1 kb. The purified PCR products were quantified, and equal amounts of immunoprecipitated samples and corresponding input samples were coupled to Cy™3 and Cy™5 dyes as described above.

The labelled samples (total amount of 3–5 µg) were hybridized onto an Agilent 4×44 K *S. pombe* Genome ChIP-on-chip microarray according to the manufacturer′s instructions (Agilent Technology, Santa Clara, CA) except for the use of Human Cot-1 DNA. The washing and scanning of the microarrays were performed as described above. The ChIP-chip data was normalized by scaling in Limma [73] and analyzed by ChIPOTle Peak Finder Excel Macro [77] with the default setting of log_2_ ratio cut-off of 1. Peaks located within 3 kb upstream of a start codon and 2 kb downstream of a start codon within a coding region or 3′-UTR, in the case of short ORFs, were assigned to the gene. ChIP-chip data sets are found in Tables S3, S5, and S7. Genes with multiple peaks are noted in the data set with the peak values. The ChIP-chip data has been submitted to the NCBI Gene Expression Omnibus Database (GSE41730).

### Motif-finding analysis

The transcription factor binding specificities were determined by RankMotif^++^ [31] and MEME [30]. *S. pombe* promoter sequences 1000 bp upstream of the translational start site were used for these motif-finding algorithms. For MEME, promoter sequences of genes with various log ratio thresholds from expression microarray and ChIP-chip experiments were input into the MEME online server. RankMotif^++^ was applied to the entire expression microarray data since its motif-searching algorithm is threshold independent. The consensus sequences of the transcription factor binding sites were displayed by submitting the position weight matrices obtained from RankMotif^++^ analysis into the enoLOGOS online server [78].

### Flocculation assays

Strains were grown in flasks at 30°C for the appropriate time, and 10 ml of culture was transferred to culture tubes for strains with larger floc sizes. Images were acquired immediately after vigorous shaking in glass culture tubes with a Canon G10 digital camera. For strains with mild flocculation, flocs were harder to visualize in culture tubes, and therefore, were observed in 90 mm plastic petri dishes. 10–15 ml of culture was transferred to petri dishes, followed by gentle shaking [8] on an orbital low-speed shaker (Labnet International, Woodridge, NJ) at maximum speed for one hour in room temperature. Floc images in petri dishes were captured using a SPImager (S&P Robotics Inc., Toronto, ON). Deflocculation of flocculent strains was performed by the addition of 2–20% D-(+)-galactose or 10 mM EDTA. The reflocculation of the deflocculated cells was performed by washing with water, resuspending the cells in YES or EMM medium or 100 mM CaCl_2_ and allowing the culture to sit for 30 min at room temperature.

For the overexpression of *pfl^+^* genes, the strains were inoculated at a concentration of 10^7^ cells in 100 ml of EMM without thiamine and cultured for 3 days at 30°C. For the weaker flocculent strains (*pfl2* ^+^–*pfl9* ^+^), 5 ml of the 3-day culture was then inoculated into 100 ml of fresh EMM without thiamine and incubated for another 3–4 days at 30°C followed by the petri dish flocculation assay as described above. Fresh EMM medium was added on the third day to prevent cells from remaining in stationary phase. Flocculation assays for the more flocculent overexpression strains were similarly carried out except the induction times were less than three days and did not require refeeding with fresh EMM medium. It should be noted that the empty vector control cells also eventually flocculate after refeeding with fresh EMM medium, but the onset of flocculation and flocs were delayed for several days and less pronounced, respectively, compared to the weakest flocculent overexpression strains. Wild-type strain and deletion mutants (*mbx2*Δ, *gsf2*Δ, *cbf12*Δ, *adn2*Δ and *adn3*Δ) were induced to flocculate by inoculating cells at a concentration of 10^8^ cells in 100 ml of YEGlycEtOH medium and culturing for 5 days at 30°C followed by the petri dish flocculation assay as described above.

### Agar adhesion and invasive growth assay

A patch of cells approximately 1/6 of a 90 mm petri dish was grown on YES medium for two days at 30°C and transferred as described in [29] onto a LNB plate (0.067 g/L yeast nitrogen base without amino acids (Bacto), 20 g/L glucose, 20 g/L agar, salts and vitamins as for EMM) with an underlying layer of YE + ALU (0.5% YE, 225 mg/L adenine, leucine, and uracil each) [79]. The plates were incubated at 30°C for 2 weeks before testing for cell-to-surface adhesion by washing cells off under a gentle stream of water and for invasive growth by rubbing the remaining cells off the agar with a finger under a stream of water. For strains showing resistance to rigorous washing by finger, a small section of the agar was cut out and observed under a Zeiss AxioScope A1 tetrad microscope (Zeiss, Thornwood, NY). Invasive growth was observed by the presence of elongated and branched cells remaining underneath the agar [29], [79].

### Fluorescence microscopy

Images of GFP-tagged cells were acquired with a Zeiss AxioScope 2 microscope (Zeiss, Thornwood, NY) and Scion CFW Monochrome CCD Firewire Camera (Scion Corporation, Frederick, MD). Fluorescence intensity was quantitated using the open source software ImageJ (version 1.44) (National Institutes of Health). First, the background signal for each image was subtracted using the “Subtract Background” function (50 pixel rolling ball radius). Individual cells were then selected as regions of interest using the freehand or polygon selection tools. Using the “Set Measurements” function both the area and integrated density were determined for each selected cell (n ranged between 27 and 50). Corrected GFP intensity was determined for each cell and was defined as the quotient of integrated density/area in background subtracted images. The averaged integrated density/area measurements for a given number cells is presented as the mean corrected GFP intensity with standard deviation. Significant differences between means were calculated by the Student *t-test*. To view nuclei and cell wall material, cells were methanol-fixed and stained with DAPI (1 µg/ml) and calcofluor white (50 µg/ml), respectively.

## Supporting Information

Figure S1 Overexpression of cell wall-remodeling genes enhances cell-to-surface adhesion, but not invasive growth. The assays for adhesion and invasive growth were carried out as described in the Materials and Methods.
(TIF)

Table S1 *Schizosaccharomyces pombe* strains used in this study.
(DOC)

Table S2 Expression Microarray Profiling of *mbx2-HA OE* strain versus empty vector strain.
(XLSX)

Table S3 *mbx2-HA* ChIP-chip data.
(XLSX)

Table S4 Expression Microarray Profiling of *rfl1* deletion strain versus isogenic wild type.
(XLSX)

Table S5 *rfl1-HA* ChIP-chip data.
(XLSX)

Table S6 Expression Microarray Profiling of *cbf12-HA OE* strain versus empty vector strain.
(XLSX)

Table S7 *cbf12-HA* ChIP-chip data.
(XLSX)

Table S8 Expression Microarray Profiling of *yox1* deletion strain versus isogenic wild type.
(XLSX)

Table S9 Expression Microarray Profiling of *sre2* deletion strain versus isogenic wild type.
(XLSX)

Table S10 Expression Microarray Profiling of *cbf11* deletion strain versus isogenic wild type.
(XLSX)

Table S11 Expression Microarray Profiling of *adn2OE* strain versus empty vector strain.
(XLSX)

Table S12 Expression Microarray Profiling of *adn3OE* strain versus empty vector strain.
(XLSX)

Table S13 Validation of putative targets and overexpressed genes by qPCR. The log_2_ ratios determined from expression microarrays are shown for comparison. Culturing, RNA extraction and reverse transcription for each strain were performed independently from the microarray experiments. Primer sets were checked for specificity by the presence of a single amplicon of the correct size using their melting curves and gel electrophoresis. The *act1^+^* gene was used as a reference for determining the relative expression of putative targets and overexpressed genes. Quantitative PCR was performed on a StepOne Real-Time PCR System with SYBR® green master mix (Life Technologies, Carlsbad, CA) and the following program: 95°C for 10 min, 40 cycles of 95°C for 15 sec and 58°C for 1 min, followed by a melting curve program of 58°C to 95°C with a heating rate of 0.3°C per second. Three replicates were carried out for each combination of query gene and strain. The relative expression of each query gene was compared between the mutant and the corresponding wild type or empty vector strain. Fold changes were determined by ΔΔCt method according to manufacturer's recommendation (Life Technologies).
(DOC)

Table S14 Degree of flocculation observed in flocculent strains. Strains were grown in EMM minus thiamine medium unless indicated. Cultures were refed with fresh medium on the third day to prevent entry into stationary phase (see Materials and Methods). To semi-quantify the amount of flocculation, 5–10 ml of culture was centrifuged (800× g, 2 min, 25°C), and deflocculated by washing once with 10 ml of 10 mM EDTA. The culture was subsequently washed three times with 15 ml of water and resuspended in water at a final concentration of ∼10^7^ cells/ml. Reflocculation was carried out by the addition of CaCl_2_ at a final concentration of 20 mM to 2.7 ml of resuspended culture in a 60 mm petri dish shaken on an orbital low-speed shaker (Labnet International, Woodridge, NJ) at maximum speed for 30 min in room temperature. The entire culture was pipetted carefully into a 3.0 ml cuvette and an OD_600_ reading close to the top of the cuvette was obtained with a Spectramax Plus microplate reader (Molecular Devices, Sunnyvale, CA). A control culture was carried out similarly except no CaCl_2_ was added. The degree of flocculation was determined by subtracting the difference of the optical density of the reflocculation culture and the control culture from 1 as described in Kobayashi O, Hayashi N, Kuroki R, Sone H, (1998) J. Bacteriol. 180: 6503–6510. The values of the degree of flocculation were derived from at least two technical replicates.
(DOC)
