# Supplementary figures and images for: Deciphering the Transcriptional-Regulatory Network of Flocculation in Schizosaccharomyces pombe
Source: PLoS Genet. 2012 Dec 6;8(12):e1003104. doi: 10.1371/journal.pgen.1003104 (PMC3516552; doi:10.1371/journal.pgen.1003104)

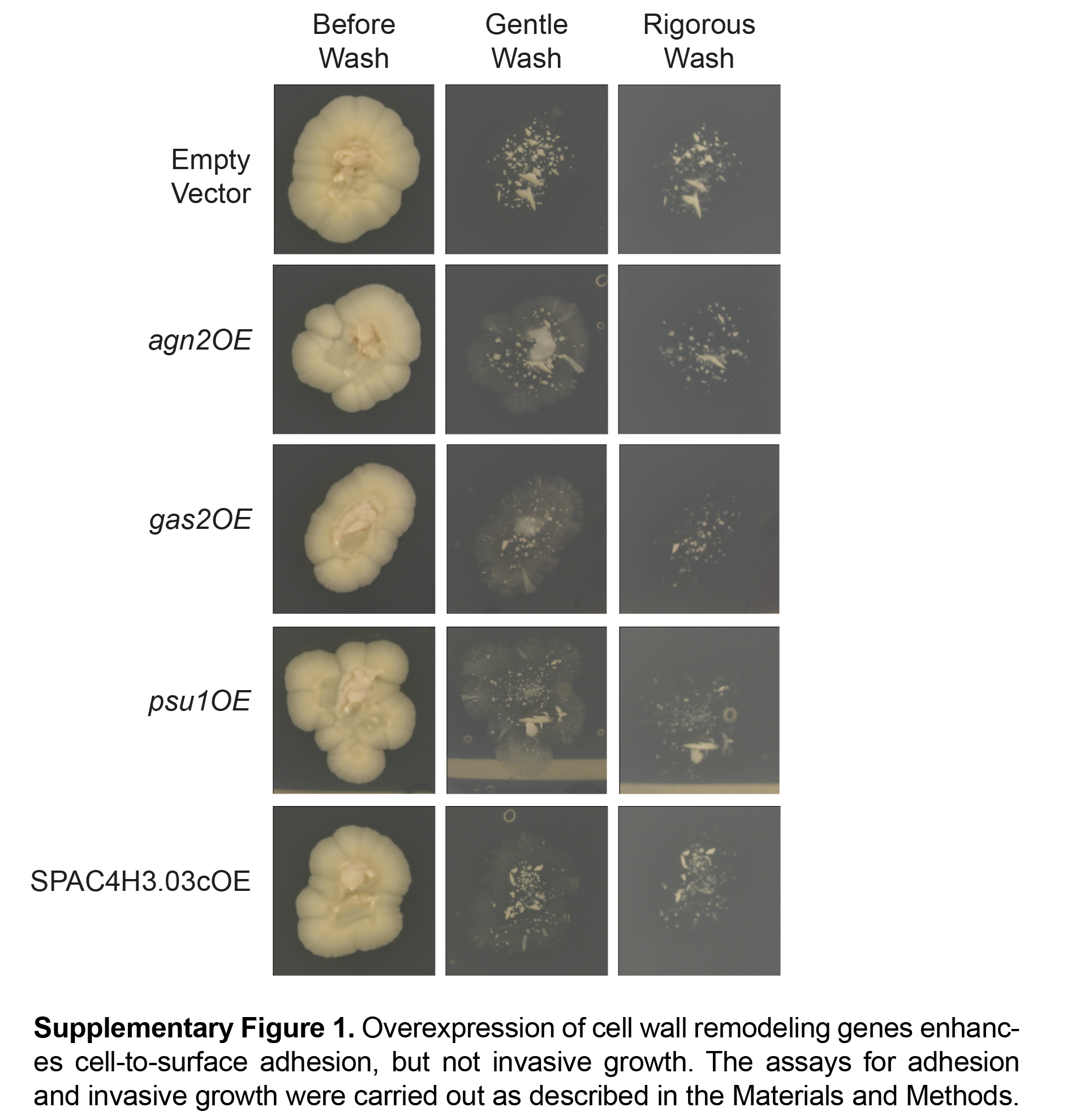

Supplement: Figure S1 — Overexpression of cell wall-remodeling genes enhances cell-to-surface adhesion, but not invasive growth. The assays for adhesion and invasive growth were carried out as described in the Materials and Methods. (TIF) [file pgen.1003104.s001.tif]
