# Supplementary material for: Deciphering the Transcriptional-Regulatory Network of Flocculation in Schizosaccharomyces pombe
Source: PLoS Genet. 2012 Dec 6;8(12):e1003104. doi: 10.1371/journal.pgen.1003104 (PMC3516552; doi:10.1371/journal.pgen.1003104)
Supplement: Table S1 — Schizosaccharomyces pombe strains used in this study. (DOC) [file pgen.1003104.s002.doc]

**Table S1**. ***Schizosaccharomyces pombe* strains used in this study**

| **Strain** | **Genotype** | **Reference** |
| --- | --- | --- |
| 972h- | *972 h-* | JK |
| JK366 | *ade6-21x leu1-32 ura4-D18 h^+^* | JK |
| JK25 | *leu1-32 h^-^* | JK |
| MBY192 | *leu1-32 ura4-D18 h^-^* | JK |
| MBY1343 | *ura4-D18 h^-^* | JK |
| GCY784 | *pSLF272 mbx2-HA ura4-D18 h^-^* | This work |
| GCY828 | *rfl1::KanMX6 h^-^* | This work |
| GCY942 | *pSLF272 ura4-D18 h^-^* | This work |
| GCY944  GCY953 | *yox1::KanMX6 h^-^*  *cbf11::KanMX6 h^-^* | This work  This work |
| GCY958 | *sre2::KanMX6 h^-^* | This work |
| GCY971 | *mbx2::KanMX6 h^-^* | This work |
| GCY1143 | *pREP1-rfl1^+^ leu1-32 h^-^* | This work |
| GCY1201 | *pREP1-mbx2^+^ leu1-32 h^-^* | This work |
| GCY1346 | *pSLF272 cbf12-HA ura4-D18 h^-^* | This work |
| GCY1444 | *pREP1 pfl3^+^ leu1-32 ura4-D18 h^-^* | This work |
| GCY1446  GCY1448 | *pREP1 fta5^+^ leu1-32 ura4-D18 h^-^*  *pREP1 pfl7^+^ leu1-32 ura4-D18 h^-^* | This work  This work |
| GCY1450 | *pREP1 pfl4^+^ leu1-32 ura4-D18 h^-^* | This work |
| GCY1452 | *pREP1 pfl5^+^ leu1-32 ura4-D18 h^-^* | This work |
| GCY1454 | *pREP1 pfl6^+^ leu1-32 ura4-D18 h^-^* | This work |
| GCY1458 | *pSFL272 rfl1-HA leu1-32 ura4-D18 h^-^* | This work |
| GCY1470 | *mbx2::NatMX6 rfl1::KanMX6 h^-^* | This work |
| GCY1640 | *gsf2::KanMX6 leu1-32 ura4-D18 h^-^* | This work |
| GCY1646 | *pREP1 pfl6::KanMX6 pfl6::NatMX6 h^+^ (SPAC977.07c::KanMX6 SPBC1348.08c::NatMX6)* | This work |
| GCY1647 | *pREP1-mbx2^+^ pfl6::KanMX6 pfl6::NatMX6 h^+^ (SPAC977.07c::KanMX6 SPBC1348.08c::NatMX6)* | This work |
| GCY1651 | *GFP-mbx2::KanMX6 leu1-32 ura4-D18 h^-^* | This work |
| GCY1652 | *pREP1-mbx2^+^-INT leu1-32 ura4-D18 h^+^* | This work |
| GCY1685 | *pREP1 pREP2 leu1-32 ura4-D18 h^-^* | This work |
| GCY1686 | *pREP1 pfl9^+^ leu1-32 ura4-D18 h^-^* | This work |
| GCY1690 | *pREP1-fta5^+^ / pREP2-pfl9^+^ leu1-32 ura4-D18 h^-^* | This work |
| GCY1691 | *pREP1-pfl4^+^ pREP2-pfl9^+^ leu1-32 ura4-D18 h^-^* | This work |
| GCY1692 | *pREP1-pfl6^+^ / pREP2-pfl9^+^ leu1-32 ura4-D18 h^-^* | This work |
| GCY1696 | *pREP1 gsf2::KanMX6 leu1-32 ura4-D18 h^-^* | This work |
| GCY1697 | *pREP1-mbx2^+^ gsf2::KanMX6 leu1-32 ura4-D18 h^-^* | This work |
| GCY1699 | *pREP1 pfl9::KanMX6 leu1-32 ura4-D18 h^+^* | This work |
| GCY1700 | *pREP1-mbx2^+^ pfl9::KanMX6 leu1-32 ura4-D18 h^+^* | This work |
| GCY1729 | *pREP1 gsf2::KanMX6 pfl9::NatMX6 leu1-32 ura4-D18 h^-^* | This work |
| GCY1730 | *pREP1-mbx2^+^gsf2::KanMX6 pfl9::NatMX6 leu1-32 ura4-D18 h^-^* | This work |
| GCY1774 | *pREP1 fta5::KanMX6 ade6-21x leu1-32 ura4-D18 h^+^* | This work |
| GCY1776 | *pREP1-mbx2^+^ fta5::KanMX6 ade6-21x leu1-32 ura4-D18 h^+^* | This work |
| GCY1778 | *pREP1 pfl7::KanMX6 leu1-32 ura4-D18 h^+^* | This work |
| GCY1780 | *pREP1-mbx2^+^ pfl7::KanMX6 leu1-32 ura4-D18 h^+^* | This work |
| GCY1782 | *pREP1 pfl4::KanMX6 ade6-21x leu1-32 ura4-D18 h^+^* | This work |
| GCY1784 | *pREP1-mbx2^+^ pfl4::KanMX6 ade6-21x leu1-32 ura4-D18 h^+^* | This work |
| GCY1786 | *pREP1 pfl5::KanMX6 ade6-21x leu1-32 ura4-D18 h^+^* | This work |
| GCY1788 | *pREP1-mbx2^+^ pfl5::KanMX6 ade6-21x leu1-32 ura4-D18 h^+^* | This work |
| GCY1798 | *pREP1 pfl3::KanMX6 leu1-32 ura4-D18 h^-^* | This work |
| GCY1800 | *pREP1-mbx2^+^ pfl3::KanMX6 leu1-32 ura4-D18 h^-^* | This work |
| GCY1802 | *pREP1 mbx2-GFP::KanMX6 ade6-21x leu1-32 ura4-D18 h^+^* | This work |
| GCY1804 | *pREP1-rfl1^+^ mbx2-GFP::KanMX ade6-21x leu1-32 ura4-D18 h^+^* | This work |
| GCY1830 | *GFP-mbx2::KanMX6 rfl1::NatMX6 leu1-32 ura4-D18 h^-^* | This work |
| GCY1843 | *KanMX6::nmt41-gsf2^+^ ade6-216 leu1-32 ura4-D18 h^+^* | This work |
| GCY1848 | *pREP1-adn2^+^ leu1-32 h^-^* | This work |
| GCY1850 | *pREP1-adn3^+^ leu1-32 h^-^* | This work |
| GCY1852 | *pREP1-adn3^+^ pREP2-adn2^+^ leu1-32 ura4-D18 h^-^* | This work |
| GCY1860 | *pREP1 pfl2^+^ leu1-32 ura4-D18 h^-^* | This work |
| GCY1871 | *pREP1 pfl2::KanMX6 leu1-32 ura4-D18 h^-^* | This work |
| GCY1872 | *pREP1-mbx2^+^ pfl2::KanMX6 leu1-32 ura4-D18 h^-^* | This work |
| GCY1874 | *pREP1 GFP-mbx2::KanMX6 leu1-32 ura4-D18 h^-^* | This work |
| GCY1875 | *pREP1-mbx2^+^ GFP-mbx2::KanMX6 leu1-32 ura4-D18 h^-^* | This work |
| GCY2037 | *pREP1 rfl1-GFP::KanMX6 ade6-21x leu1-32 ura4-D18 h^+^* | This work |
| GCY2039 | *pREP1-rfl1^+^ rfl1-GFP::KanMX6 ade6-21x leu1-32 ura4-D18 h^+^* | This work |
| GCY2088 | *pREP1 leu1-32 h^-^* | This work |
| GCY2112 | *pREP1-cbf12^+^ leu1-32 h^-^* | This work |
| GCY2128 | *rfl1::KanMX6 gsf2::KanMX6 pfl9::NatMX6 h^X^* | This work |
| GCY2129 | *rfl1::KanMX6 gsf2::KanMX6 h^X^* | This work |
| GCY2130 | *rfl1::KanMX6 pfl9::NatMX6 h^X^* | This work |
| GCY2143 | *pREP1-mbx2^+^-INT pREP2 leu1-32 ura4-D18 h^-^* | This work |
| GCY2144 | *pREP1-mbx2^+^-INT pREP2-rfl1^+^* *leu1-32 ura4-D18 h^-^* | This work |
| GCY2175 | *yox1::NatMX6 gsf2::KanMX6 h^X^* | This work |
| GCY2179 | *cbf11::NatMX6 gsf2::KanMX6 h^X^* | This work |
| GCY2265 | *sre2::NatMX6 gsf2::KanMX6 h^X^* | This work |
| GCY2388 | *pREP1-adn2^+^ gsf2::KanMX6 leu1-32 ura4-D18 h^-^* | This work |
| GCY2390 | *pREP1-adn3^+^ gsf2::KanMX6 leu1-32 ura4-D18 h^-^* | This work |
| GCY2396 | *pREP2-agn2^+^ leu1-32 ura4-D18 h^-^* | This work |
| GCY2398 | *pREP2-gas2^+^ leu1-32 ura4-D18 h^-^* | This work |
| GCY2400 | *pREP2-psu1^+^ leu1-32 ura4-D18 h^-^* | This work |
| GCY2402 | *pREP2-SPAC4H3.03^+^ leu1-32 ura4-D18 h^-^* | This work |
| GCY2429 | *pREP1 cbf12::GFP-KanMX6 leu1-32 h^-^* | This work |
| GCY2431 | *pREP1-cbf12^+^ cbf12::GFP-KanMX6 leu1-32 h^-^* | This work |
| Bioneer | *adn2::KanMX4 ade6-21x leu1-32 ura4-D18 h^+^* | Bioneer |
| Bioneer | *adn3::KanMX4 ade6-21x leu1-32 ura4-D18 h^+^* | Bioneer |
|  |  |  |
