# Supplementary material for: Deciphering the Transcriptional-Regulatory Network of Flocculation in Schizosaccharomyces pombe
Source: PLoS Genet. 2012 Dec 6;8(12):e1003104. doi: 10.1371/journal.pgen.1003104 (PMC3516552; doi:10.1371/journal.pgen.1003104)
Supplement: Table S13 — Validation of putative targets and overexpressed genes by qPCR. The log2 ratios determined from expression microarrays are shown for comparison. Culturing, RNA extraction and reverse transcription for each strain were performed independently from the microarray experiments. Primer sets were checked for specificity by the presence of a single amplicon of the correct size using their melting curves and gel electrophoresis. The act1+ gene was used as a reference for determining the relative expression of putative targets and overexpressed genes. Quantitative PCR was performed on a StepOne Real-Time PCR System with SYBR® green master mix (Life Technologies, Carlsbad, CA) and the following program: 95°C for 10 min, 40 cycles of 95°C for 15 sec and 58°C for 1 min, followed by a melting curve program of 58°C to 95°C with a heating rate of 0.3°C per second. Three replicates were carried out for each combination of query gene and strain. The relative expression of each query gene was compared between the mutant and the corresponding wild type or empty vector strain. Fold changes were determined by ΔΔCt method according to manufacturer's recommendation (Life Technologies). (DOC) [file pgen.1003104.s014.doc]

**Table S13.** **Validation of putative targets and overexpressed genes by qPCR. The log_2_ ratios determined from expression microarrays are shown for comparison.**

| **Strain** | **ORF** | **qPCR (log2 fold change)** | **Microarray (log2 fold change)** |
| --- | --- | --- | --- |
| *cbf12OE* | *gsf2^+^* | 6.7 | 4.2 |
| *rfl1OE/Rfl1-GFP* | *rfl1-GFP* | 0.2 | N/A |
| *mbx2OE* | *mbx2^+^* | 9.4 | 3.7 |
| *mbx2OE* | *gsf2^+^* | 4.3 | 6.4 |
| *mbx2OE* | *pfl4^+^* | 2.4 | 5.4 |
| *mbx2OE* | *pfl6^+^* | 8.1 | 6.1 |
| *mbx2OE* | *fta5^+^* | 1.6 | 5.3 |
| *mbx2OE* | *pfl9^+^* | 9.2 | 6.8 |
| *rfl1*∆ | *gsf2^+^* | 4.2 | 5.9 |
| *rfl1*∆ | *pfl4^+^* | 1.9 | 3.9 |
| *rfl1*∆ | *pfl6^+^* | 2.2 | 2.9 |
| *rfl1*∆ | *fta5^+^* | 3.5 | 4.0 |
| *rfl1*∆ | *pfl9^+^* | 5.0 | 6.3 |
| *pfl2OE* | *pfl2^+^* | 6.1 | N/A |
| *pfl3OE* | *pfl3^+^* | 10.9 | N/A |
| *pfl4OE* | *pfl4^+^* | 10.3 | N/A |
| *pfl5OE* | *pfl5^+^* | 10.1 | N/A |
| *pfl6OE* | *pfl6^+^* | 16.2 | N/A |
| *pfl7OE* | *pfl7^+^* | 16.6 | N/A |
| *adn2OE* | *adn2^+^* | 7.3 | 6.9 |
| *adn3OE* | *adn3^+^* | 7.2 | 6.4 |
| *agn2OE* | *agn2^+^* | 9.2 | N/A |
| *SPAC4H3.03OE* | *SPAC4H3.03c* | 10.9 | N/A |
| *psu1OE* | *psu1^+^* | 5.5 | N/A |
| *gas2OE* | *gas2^+^* | 5.3 | N/A |
| *adn2OE* | *gas2^+^* | 2.7 | 4.2 |
| *adn2OE* | *SPAC4H3.03c* | 3.5 | 5.2 |
| *adn2OE* | *psu1^+^* | -0.6 | -0.53 |
| *adn3OE* | *gas2^+^* | 3.3 | 2.9 |
| *adn3OE* | *SPAC4H3.03c* | 1.6 | 1.1 |
| *adn3OE* | *psu1^+^* | 0.1 | 1.0 |
