# Supplementary material for: Deciphering the Transcriptional-Regulatory Network of Flocculation in Schizosaccharomyces pombe
Source: PLoS Genet. 2012 Dec 6;8(12):e1003104. doi: 10.1371/journal.pgen.1003104 (PMC3516552; doi:10.1371/journal.pgen.1003104)
Supplement: Table S14 — Degree of flocculation observed in flocculent strains. Strains were grown in EMM minus thiamine medium unless indicated. Cultures were refed with fresh medium on the third day to prevent entry into stationary phase (see Materials and Methods). To semi-quantify the amount of flocculation, 5–10 ml of culture was centrifuged (800× g, 2 min, 25°C), and deflocculated by washing once with 10 ml of 10 mM EDTA. The culture was subsequently washed three times with 15 ml of water and resuspended in water at a final concentration of ∼107 cells/ml. Reflocculation was carried out by the addition of CaCl2 at a final concentration of 20 mM to 2.7 ml of resuspended culture in a 60 mm petri dish shaken on an orbital low-speed shaker (Labnet International, Woodridge, NJ) at maximum speed for 30 min in room temperature. The entire culture was pipetted carefully into a 3.0 ml cuvette and an OD600 reading close to the top of the cuvette was obtained with a Spectramax Plus microplate reader (Molecular Devices, Sunnyvale, CA). A control culture was carried out similarly except no CaCl2 was added. The degree of flocculation was determined by subtracting the difference of the optical density of the reflocculation culture and the control culture from 1 as described in Kobayashi O, Hayashi N, Kuroki R, Sone H, (1998) J. Bacteriol. 180: 6503–6510. The values of the degree of flocculation were derived from at least two technical replicates. (DOC) [file pgen.1003104.s015.doc]

**Table S14**. **Degree of flocculation observed in flocculent strains.**

| **Strain** | **Incubation (days)** | **Degree of Flocculation** |
| --- | --- | --- |
| *WT* (YES medium) | 5 days | 0.00 +/- 0.14 |
| *WT* (Glycerol-inducing medium) | 5 days | 71.51 +/- 3.47 |
| *rfl1*∆ (YES medium) | 2 days | 74.31 +/- 0.56 |
| *mbx2OE* | 1 day | 81.81 +/- 2.10 |
| *mbx2OE gsf2*∆ | 1 day | 5.17 +/- 1.16 |
| *mbx2OE pfl2*∆ | 1 day | 76.67 +/- 2.36 |
| *mbx2OE pfl3*∆ | 1 day | 76.33 +/- 2.76 |
| *mbx2OE pfl4*∆ | 1 day | 78.13 +/- 4.96 |
| *mbx2OE pfl5*∆ | 1 day | 82.30 +/- 1.38 |
| *mbx2OE pfl7*∆ | 1 day | 81.64 +/- 0.57 |
| *mbx2OE fta5*∆ | 1 day | 76.28 +/- 1.46 |
| *mbx2OE pfl9*∆ | 1 day | 80.74 +/- 1.83 |
| *mbx2OE gsf2*∆ *pfl9∆* | 1 day | 5.85 +/-0.02 |
| *gsf2OE* | 2 days | 84.85 +/- 0.90 |
| *pfl2OE* | 4 days | 25.17 +/- 0.09 |
| *pfl3OE* | 6 days | 83.42 +/- 0.46 |
| *pfl4OE* | 6 days | 62.75 +/- 1.32 |
| *pfl5OE* | 6 days | 84.52 +/- 1.01 |
| *pfl6OE* | 6 days | 54.88 +/- 1.46 |
| *pfl7OE* | 6 days | 8.00 +/- 2.38 |
| *fta5OE* | 6 days | 11.48 +/- 1.00 |
| *pfl9OE* | 6 days | 3.89 +/- 1.36 |
| *pfl4OE pfl9OE* | 4 days | 59.37 +/- 1.60 |
| *pfl6OE pfl9OE* | 4 days | 32.53 +/- 3.56 |
| *fta5OE pfl9OE* | 4 days | 43.46 +/- 1.54 |
| *empty vector* | 6 days | 5.19 +/- 2.19 |
| *cbf12OE* | 2 days | 77.13 +/- 4.31 |
| *cbf12OE gsf2∆* | 2 days | 2.14 +/- 1.03 |
| *cbf12OE pfl7∆* | 2 days | 76.33 +/- 0.90 |
| *cbf11∆* (YES medium) | 2 days | 28.20 +/- 6.46 |
| *sre2∆* (YES medium) | 2 days | 57.49 +/- 2.51 |
| *yox1∆* (YES medium) | 2 days | 28.27 +/- 0.45 |
| *cbf11∆ gsf2∆* (YES medium) | 2 days | 19.11 +/- 5.37 |
| *sre2∆ gsf2∆* (YES medium) | 2 days | 7.59 +/- 1.10 |
| *yox1∆ gsf2∆* (YES medium) | 2 days | 9.65 +/- 1.24 |
| *adn2OE* | 4 days | 24.68 +/- 0.85 |
| *adn3OE* | 4 days | 33.61 +/- 2.83 |
| *adn2OE gsf2∆* | 4 days | 7.81 +/- 2.52 |
| *adn3OE gsf2∆* | 4 days | 2.20 +/- 2.68 |
| *agn2OE* | 4 days | 91.38 +/- 0.14 |
| *gas2OE* | 4 days | 80.55 +/- 7.76 |
| *psu1OE* | 4 days | 82.13 +/- 0.91 |
| *SPAC4H3.03OE* | 4 days | 79.41 +/- 4.29 |
| *GFP-mbx2* (Glycerol-inducing medium) | 5 days | 36.02 +/- 5.74 |
